# Adjoint *L*-functions, congruence ideals, and Selmer groups over $\textrm{GL}_n$

**DOI:** 10.1007/s40993-026-00721-6

**Published:** 2026-02-27

**Authors:** Ho Leung Fong

**Affiliations:** School of Mathematical and Physical Sciences, University of Sheffield, Sheffield, United Kingdom

**Keywords:** *L*-functions, Congruence, Cohomology, Selmer groups

## Abstract

The study of special values of adjoint *L*-functions and congruence ideals is gradually becoming a classical theme in number theory, driven by the Bloch-Kato conjecture and generalisations of Wiles-Lenstra’s numerical criterion. In this paper, we relate $L(1,\pi ,{{\,\textrm{Ad}\,}}^\circ )$ to the congruence ideals for cohomological cuspidal automorphic representations $\pi$ of $\textrm{GL}_n$ over any number field. We then use this result to deduce relationships between the congruences of automorphic forms and adjoint *L*-functions. For CM fields, using the existence of Galois representations, we apply the result to obtain a lower bound on the cardinality of certain Selmer groups in terms of $L(1,\pi ,{{\,\textrm{Ad}\,}}^\circ )$. This can be viewed as partial progress on the Bloch-Kato conjecture. The main technical ingredients are a careful study of the cohomology associated with the locally symmetric space of $\textrm{GL}_n$, its relation to automorphic representations, and the establishment of some algebraic properties of the congruence ideals. We anticipate that the methods developed here will find further applications in related problems, particularly in the study of congruence modules and their relation to the arithmetic of automorphic forms.

## Introduction

In number theory, we study many different types of *L*-functions, such as *L*-functions of elliptic curves, Artin *L*-functions, and Dirichlet L-functions. A recurring theme is that the special values of these *L*-functions often encode deep arithmetic information. Some notable examples include the analytic class number formula, the Herbrand-Ribet theorem, and the Birch-Swinnerton-Dyer conjecture.

The focus of this paper is the adjoint *L*-function of an automorphic representation. Special values of adjoint *L*-functions frequently reflect congruences between automorphic forms, and these congruences can in turn be measured by the so-called congruence ideal. The relationship between adjoint *L*-values and congruence ideals has by now become a central theme in modern number theory; for a historical overview, we refer the reader to the introduction of [1]. Here we offer a complementary motivation.

In 1994, Andrew Wiles and Richard Taylor proved Fermat’s last theorem by showing that every semistable elliptic curve is modular. The key is to establish a modularity lifting theorem, which in their setup boils down to showing that the universal deformation ring *R* of some residual Galois representation is isomorphic to a suitable Hecke algebra *T*. To do so, they used the Taylor-Wiles method to prove a modularity lifting theorem in the minimally ramified case [2]. To extend the theorem to the non-minimal case, Wiles [3] used a numerical criterion for ring isomorphism. To apply the criterion involves studying the tangent space of *R* on the one hand and examining an invariant called the congruence ideal $\eta _T$ of *T* on the other hand.

It has since proved fruitful to study congruence ideals not only for Hecke algebras but also for various cohomology modules. In [4], the congruence ideals associated with the cohomology of certain locally symmetric spaces are related to the value at $s=1$ of the adjoint *L*-function for $\textrm{GL}_2$ over specific number fields. Using the work of [1], we generalize these results to $\textrm{GL}_n$ over an arbitrary number field.

Let $\pi$ be a regular algebraic cuspidal automorphic representation of $\textrm{GL}_n(\mathbb {A}_F)$, where *F* is a number field. Our first main result is Theorem 4.14, which establishes that, under suitable assumptions, the (cohomological) congruence ideal of $\pi$ is generated by a special value of the normalised adjoint *L*-function $L^{alg}(1,\pi ,{{\,\textrm{Ad}\,}}^0,\epsilon )$.

Building on Theorem 4.14, we derive consequences for congruences between automorphic forms. In Corollaries 4.17 and 4.20, we show that under suitable assumptions, if the *p*-adic valuation of $L(1,\pi ,{{\,\textrm{Ad}\,}}^\circ ,\epsilon )$ is positive, then there is an automorphic representation $\pi '\not \cong \pi$ whose Hecke eigensystem is congruent to that of $\pi$.

In the last subsection, we provide in Theorem 4.26 a lower bound for the cardinality of a certain Selmer group in terms of special values of *L*-functions when *F* is a *CM* field. This can be viewed as partial progress on the Bloch-Kato conjecture.

### Strategy

We briefly summarise the strategy of the paper, omitting certain technical details in order to emphasise the main ideas.

The starting point is to realise automorphic representations in cohomology and to work at the level of cohomology. By results of Borel and Franke [5, 6], any regular algebraic cuspidal automorphic representation of $\textrm{GL}_n(\mathbb {A}_F)$ contributes to the cohomology of the locally symmetric space $X_U$ associated with $\textrm{GL}_n$. Poincaré duality furnishes a perfect pairing between the singular cohomology and the compactly supported cohomology of $X_U$ with $\mathbb {Z}_p$-coefficients. Under the additional assumption that the cohomology of the boundary of $X_U$ is *p*-torsion free, this pairing induces a perfect pairing on the inner cohomology of $X_U$.

By Lemma 3.5, any such perfect pairing that is suitably compatible with the action of the Hecke algebra yields an explicit formula for the corresponding congruence ideal: the ideal is generated by the value of the pairing on appropriate $\mathbb {Z}_p$-generators. In our setting, the relevant pairing is the cup product on cohomology, which, on the automorphic side, corresponds essentially to the Petersson inner product of two automorphic forms. Via the Rankin-Selberg method, this inner product can be expressed in terms of the adjoint *L*-function. This leads directly to a formula for the congruence ideal in terms of the value $L(1,\pi ,{{\,\textrm{Ad}\,}}^0,\epsilon )$ in Theorem 4.14.

Using the relationship between congruence ideals and congruences of automorphic representations (Lemma 3.6), we then obtain criteria for the existence of congruent automorphic representations in terms of adjoint *L*-values (Corollary 4.17). Finally, invoking the local-global compatibility results for Galois representations established in [7], together with standard techniques from Galois deformation theory, we derive in section 4.5 (when *F* is a CM field) a lower bound for the order of a certain Selmer group in terms of special values of adjoint *L*-functions.

### Notation

A CM field means a totally imaginary quadratic extension of a totally real number field.

For a number field *F*, we write $\mathbb {A}_F$ for the ring of adeles, $F_\infty :=F\otimes _\mathbb {Q}\mathbb {R}=\prod _{v\mid \infty } F_v,$

$$\begin{aligned} \mathbb {A}_F^\infty \end{aligned}$$

the ring of finite adeles, and $\mathbb {A}_F^{\infty ,S}$ for the ring of finite adeles without the components at *S*. The contragredient of an automorphic representation of $\textrm{GL}_n(\mathbb {A}_F)$ is denoted by $\tilde{\pi }$.

If *G* is a locally profinite group and *U* is an open compact subgroup of *G*, then we let

$$\begin{aligned} \mathcal {H}(G,U)&:=\{U\text {-biinvariant compactly supported functions }G\rightarrow \mathbb {Z}\}\\&\cong \mathbb {Z}[U\backslash G/U], \end{aligned}$$

where multiplication is given by convolution with respect to the Haar measure on *G* which gives *U* volume 1. If *F* is a number field and $\mathcal {O}$ is the ring of integers of a finite extension of $\mathbb {Q}_p$, then

$$\begin{aligned} \mathbb {T}^S:= \mathcal {H}(\textrm{GL}_n(\mathbb {A}_F^{\infty ,S}),\prod _{v\notin S\cup \{\infty \}}\textrm{GL}_n(\mathcal {O}_{F_v}))\otimes _\mathbb {Z}\mathcal {O}. \end{aligned}$$

(This of course depends on $F, \mathcal {O}$.)

We write ${{\,\textrm{Ad}\,}}^0$ for the (trace zero) adjoint representation, which is the representation of $\textrm{GL}_n(\mathbb {C})$ on the $n\times n$ trace zero matrices by conjugation.

All *L*-functions include the *L*-factors at infinity unless otherwise stated.

If *F* is a number field and $G=\textrm{GL}_{n/F}$, then $K_\infty$ will mean the product of $A_G:=\mathbb {R}_{>0}$^1^and the standard maximal compact subgroup of $G(F\otimes _\mathbb {Q}\mathbb {R})$, so

$$\begin{aligned} K_\infty \cong \mathbb {R}_{>0} \cdot (O_n(\mathbb {R})^{r_1}\times U_n(\mathbb {R})^{r_2}), \end{aligned}$$

where $r_1,r_2$ are the number of real and complex places of *F* respectively and $U_n(\mathbb {R}):=\{g\in \textrm{GL}_n(\mathbb {C}): \bar{g}^T g=1\}$ is the unitary group. Also, $\mathfrak g$ will be the Lie algebra of $\textrm{GL}_n(F_\infty )$.

## Cohomology

Fix a number field *F* with $r_1$ real places and $r_2$ complex places for the entire section. Readers not very familiar with the relationship between automorphic representations and cohomology are recommended to read the excellent papers [8, 9].

### Cohomology and Hecke operators

We shall mostly follow [10, section 6] and [11, section 2.1] to define the cohomology group and Hecke operators. Most of the material is standard, but there are many different variations, so we think it is necessary to state clearly our conventions.

Let $G=\textrm{GL}_n$. For an open compact subgroup $U\subset G(\mathbb {A}_F^\infty )$, we define^2^

$$\begin{aligned} X_U:=G(F)\backslash G(\mathbb {A}_F)/K_{\infty }^\circ U. \end{aligned}$$

and

$$\begin{aligned} X:= G(F_\infty )/K_{\infty }^\circ . \end{aligned}$$

Note that $$X_U=G(F)\backslash (X\times G(\mathbb {A}_F^\infty )/U).$$

We call an element $g=(g_v)_v\in G(\mathbb {A}^\infty _F)$ *neat* if $\cap _v \Gamma _v=\{1\}$, where $\Gamma _v\subset \bar{\mathbb {Q}}^\times$ is the torsion subgroup of the subgroup generated by the eigenvalues of $g_v$. We call an open compact subgroup $K\subset G(\mathbb {A}^\infty _F)$ *neat* if all of its elements are neat.

#### Definition 2.1

We call $U\subset G(\mathbb {A}_F^\infty )$ a *good subgroup* if it is a neat subgroup of the form $U=\prod _v U_v\subset \prod \textrm{GL}_n(\mathcal {O}_{F_v})$.

Let *U* be a good subgroup and *M* be a $\mathbb {Z}[U]$-module. We define a locally constant sheaf $\mathcal {L}_M$ on $X_U$ as the sheaf of continuous sections of the map

$$\begin{aligned} G(F)\backslash (X\times G(\mathbb {A}_F^\infty )\times M)/U \rightarrow X_U \end{aligned}$$

where $G(F)\times U$ acts on $X\times G(\mathbb {A}_F^\infty )\times M$ by $(\gamma ,u)(x,g,m)=(\gamma x,\gamma g u^{-1},um)$ and *M* is equipped with the discrete topology. We define

$$\begin{aligned} H^*(X_U,M):=H^*(X_U,\mathcal {L}_M) \end{aligned}$$

to be the sheaf cohomology.

#### Proposition 2.2

[10, Proposition 6.2] If *U* is a neat^3^ subgroup, then

$$\begin{aligned} H^*(X_U,M)\cong H^*(C_{\mathbb {A}}^{\bullet }(U,M)), \end{aligned}$$

where

$$\begin{aligned} C_{\mathbb {A}}^\bullet (U,M):={{\,\textrm{Hom}\,}}_{G(F)\times U}(C_{{\mathbb {A}},\bullet }, M) \end{aligned}$$

and $C_{{\mathbb {A}},\bullet }$ is the group of singular chains on $X\times G(\mathbb {A}_F^\infty )$ with $\mathbb {Z}$ coefficients.

To define the action of the Hecke algebra, we suppose *M* is actually a left $\mathbb {Z}[\Delta ]$-module, where $\Delta \subset G(\mathbb {A}_F^\infty )$ is a submonoid containing *U*. Note that the compactness of *U* implies that $U\subset \Delta$ is a Hecke pair. Let $\mathcal {H}(\Delta ,U)$ be the set of locally constant, compactly supported functions $f:\Delta \rightarrow \mathbb {Z}$ which is *U*-biinvariant. We can (and will) regard it as a subalgebra of $\mathcal {H}(G(\mathbb {A}^\infty _F),U)$.

For $\delta \in \Delta$, let the characteristic function $[U\delta U]$ acts on the complex $C_{\mathbb {A}}^\bullet (U,M)$ by

$$\begin{aligned} ([U\delta U]^* \phi )(\sigma )=\sum \delta _i\phi (\delta _i^{-1}\sigma ) \end{aligned}$$

where $U \delta U=\bigsqcup _i \delta _i U$, $\phi \in C_{\mathbb {A}}^\bullet (U,M)$ and $\sigma \in C_{{\mathbb {A}},\bullet }.$ This is independent of the choices of $\delta _i$. By taking cohomology, we get an action of $\mathcal {H}(\Delta ,U)$ on $H^*(X_U,M)$.

Let $T_n$ and $B_n$ be the standard diagonal torus and Borel subgroup of $\textrm{GL}_{n/\mathbb {Z}}$. Let $w_0$ be the longest element of the Weyl group^4^.

#### Definition 2.3

[13, Definition 2.1] Let *A* be a commutative ring. If $\lambda \in \mathbb {Z}^n$ is a dominant weight for $\textrm{GL}_n$, then we define the algebraic representation $Ind^{\textrm{GL}_n}_{B_n}(w_0\lambda )_{/A}$ of $\textrm{GL}_{n/A}$ to be

$$\begin{aligned} \{f\in A[\textrm{GL}_n]: f(bg)=(w_0\lambda )(b) f(g) \text { for all }A\text {-algebras }B, g\in \textrm{GL}_n(B), b\in B_n(B)\} \end{aligned}$$

where $A[\textrm{GL}_n]:=Mor_{{{\,\textrm{Spec}\,}}A}(\textrm{GL}_{n/A},\mathbb {A}^1_{A})$^5^and $\textrm{GL}_{n/A}$ acts by right translation. We let

$$\begin{aligned} M_{\lambda ,A}:= Ind^{\textrm{GL}_n}_{B_n}(w_0\lambda )_{/A}(A), \end{aligned}$$

which is a representation of $\textrm{GL}_n(A)$.

If *E* is a *p*-adic field with ring of integers $\mathcal {O}$, then from [13, page 1349], $M_{\lambda ,\mathcal {O}}$ is finite free over $\mathcal {O}$. Also, $M_{\lambda ,\mathcal {O}}\otimes _{\mathcal {O}} E=M_{\lambda ,E}$ is the algebraic representation of $\textrm{GL}_n(E)$ of highest weight $\lambda$. By [14, page 19], for all $\mathcal {O}$-algebras *R*, the natural map $M_{\lambda ,\mathcal {O}}\otimes _{\mathcal {O}} R \rightarrow M_{\lambda ,R}$ is an isomorphism.

We write $\mathbb {Z}^n_+:=\{(\lambda _1,\dots ,\lambda _n)\in \mathbb {Z}^n:\lambda _1\ge \dots \ge \lambda _n\}$. Let *E* be a finite extension of $\mathbb {Q}_p$ inside $\overline{\mathbb {Q}}_p$ which contains all embeddings of *F* to $\overline{\mathbb {Q}}_p,$ $\mathcal {O}$ be the ring of integers of *E*, and $\mu \in (\mathbb {Z}^n_+)^{{{\,\textrm{Hom}\,}}(F,E)}$.

We define the $\mathcal {O}$-module

$$\begin{aligned} M_{\mu }:=M_{\mu ,\mathcal {O}}:=\bigotimes _{\tau \in {{\,\textrm{Hom}\,}}(F,E)} M_{\mu _\tau ,\mathcal {O}} \end{aligned}$$

which receives an action of $\prod _{v\mid p}\textrm{GL}_n(\mathcal {O}_{F_v})$ by $(g_v)_v \cdot \otimes m_\tau = \otimes g_{v(\tau )}m_{\tau },$ where $v(\tau )$ is the place of *F* induced by $\tau .$ Then $\textrm{GL}_n(\mathbb {A}_F^{\infty ,p})\times U_p$ acts on $M_{\mu ,\mathcal {O}}$ by projection to $U_p:=\prod _{v\mid p}U_v$. By the formalism above^6^, $\mathcal {H}(\textrm{GL}_n(\mathbb {A}_F^{\infty ,p}),U^p)$ acts on $H^*(X_U,M_{\mu ,\mathcal {O}})$.

We define $M_{\mu ,E}:=\otimes _{\tau \in {{\,\textrm{Hom}\,}}(F,E)} M_{\mu _\tau ,E}$ as an $E[\prod _{v\mid p}\textrm{GL}_n(F_v)]$-module. Then $\textrm{GL}_n(\mathbb {A}_F^{\infty })$ acts on $M_{\mu ,E}$ by projection to $\textrm{GL}_n(F_p):=\textrm{GL}_n(\prod _{v\mid p}F_v)$. By the formalism above, $\mathcal {H}(\textrm{GL}_n(\mathbb {A}_F^{\infty }),U)$ acts on $H^*(X_U,M_{\mu ,E})$. This is compatible with the construction above, i.e. we have an isomorphism $H^*(X_U,M_{\mu ,\mathcal {O}})\otimes _\mathcal {O}E \cong H^*(X_U,M_{\mu ,E})$ that is Hecke-equivariant for the restriction map $\mathcal {H}(\textrm{GL}_n(\mathbb {A}_F^{\infty ,p}),U^p) \rightarrow \mathcal {H}(\textrm{GL}_n(\mathbb {A}_F^{\infty }),U)$.

Fix an isomorphism $\iota :\overline{\mathbb {Q}}_p\xrightarrow {\sim }\mathbb {C}$ for the rest of this section. We define $M_{\mu ,\mathbb {C}}:=\otimes _{\tau \in {{\,\textrm{Hom}\,}}(F,E)} M_{\mu _\tau ,\mathbb {C}}$. This is acted on by $\prod _{v\mid p}\textrm{GL}_n(F_v)$ via $\iota$ and also by $G(F_\infty )$, where $F_\infty :=F\otimes _\mathbb {Q}\mathbb {R},$ by

$$\begin{aligned} G(F_\infty )\hookrightarrow G(F\otimes _\mathbb {Q}\mathbb {C})=\prod _{\tau \in {{\,\textrm{Hom}\,}}(F,\mathbb {C})} G(\mathbb {C})\curvearrowright \otimes _{\tau \in {{\,\textrm{Hom}\,}}(F,E)} M_{\lambda _\tau ,\mathbb {C}} \end{aligned}$$

using the identification ${{\,\textrm{Hom}\,}}(F,E)= {{\,\textrm{Hom}\,}}(F,\mathbb {C})$ given by $\iota$. Note that the 2 actions of *G*(*F*) on $M_{\mu ,\mathbb {C}}$ agree. As $M_{\mu ,\mathbb {C}}$ is an irreducible representation of $\textrm{GL}_n(F\otimes _\mathbb {Q}\mathbb {C})$, it has a central character. In particular, $A_G$ acts^7^ by a character

$$\begin{aligned} \chi ^{-1}:A_G\rightarrow \mathbb {C}^\times \end{aligned}$$

on $M_{\mu ,\mathbb {C}}.$ One can show that for all good subgroup $U\le G(\mathbb {A}_F^\infty )$,

$$\begin{aligned} H^*(X_U,M_{\mu ,\mathbb {C}})\cong H^*(\mathfrak g,K_\infty ^\circ ,C^\infty (G(F)\backslash G(\mathbb {A}_F)/U,\chi )\otimes _\mathbb {C}M_{\mu ,\mathbb {C}}), \end{aligned}$$

where $$\mathfrak g=Lie(G(F_\infty ))$$ and

$$\begin{aligned} C^\infty (G(F)\backslash G(\mathbb {A}_F)/U,\chi ) \end{aligned}$$

is the set of smooth functions $f:G(F)\backslash G(\mathbb {A}_F)/U \rightarrow \mathbb {C}$ with $f(ag)=\chi (a)f(g)$ for all $a\in A_G$. The algebra $\mathcal {H}(\textrm{GL}_n(\mathbb {A}_F^{\infty }),U)$ acts on $C^\infty (G(F)\backslash G(\mathbb {A}_F)/U,\chi )$ and this induces the Hecke action on the relative Lie algebra cohomology. Then this isomorphism of cohomology is compatible with the action of $\mathcal {H}(\textrm{GL}_n(\mathbb {A}_F^{\infty }),U)$ on both sides.

### Regular algebraic automorphic representations

Let $G=\textrm{GL}_n$ as above.

#### Definition 2.4

Let $\chi :A_G \rightarrow \mathbb {C}^\times$ be a continuous homomorphism. We write

$$\begin{aligned} L^2(G(F)\backslash G(\mathbb {A}_F),\chi ) \end{aligned}$$

for the space of measurable functions $f:G(F)\backslash G(\mathbb {A}_F) \rightarrow \mathbb {C}$ such that $f(ag)=\chi (a)f(g)$ for all $a\in A_G$ and

$$\begin{aligned} \int _{G(F)\backslash G(\mathbb {A}_F)^1}|f(g)|^2 dg<\infty , \end{aligned}$$

where

$$\begin{aligned} G(\mathbb {A}_F)^1:=\{g\in G(\mathbb {A}_F):|\det (g)|_{\mathbb {A}_F}=1\} \end{aligned}$$

and functions which agree almost everywhere are identified. Let

$$\begin{aligned} L^2_0(G(F)\backslash G(\mathbb {A}_F),\chi ) \end{aligned}$$

be the subspace of cuspidal functions in $L^2(G(F)\backslash G(\mathbb {A}_F),\chi )$, i.e. elements *f* of $L^2(G(F)\backslash G(\mathbb {A}_F),\chi )$ such that for the unipotent radical $U_P$ of every proper parabolic subgroup *P*,

$$\begin{aligned} \int _{U_P(F)\backslash U_P(\mathbb {A}_F)} f(ug) du=0 \end{aligned}$$

for almost all $g\in G(\mathbb {A}_F)^1$. We define a *cuspidal automorphic representation of character* $\chi$ to be an irreducible subrepresentation in $L^2_0(G(F)\backslash G(\mathbb {A}_F),\chi )$ of $G(\mathbb {A}_F)$. A *cuspidal automorphic representation* is one such representation for some $\chi$.

#### Definition 2.5

Let $\chi :A_G \rightarrow \mathbb {C}^\times$ be a continuous homomorphism. We let

$$\begin{aligned} L^2_d(G(F)\backslash G(\mathbb {A}_F),\chi ) \end{aligned}$$

be the discrete spectrum, i.e. the closure of the sum of all irreducible subrepresentations of $L^2(G(F)\backslash G(\mathbb {A}_F),\chi )$. We define a *discrete automorphic representation of character* $\chi$ to be an irreducible subrepresentation in $L^2_d(G(F)\backslash G(\mathbb {A}_F),\chi )$ of $G(\mathbb {A}_F)$. A *discrete automorphic representation* is one such representation for some $\chi$.

#### Remark 2.6

Note that every cuspidal automorphic representation is discrete. Also, by our definition, every discrete automorphic representation $\pi$ is a unitary Hilbert space representation of $G(\mathbb {A}_F)$ after twisting by a suitable character.^8^ It follows from the irreducibility of $\pi$ that $\pi$ has a central character. Moreover, $\pi _\infty$ is admissible by a result of Harish-Chandra [15, Theorem 4.4.5]

#### Definition 2.7

Let $\lambda \in (\mathbb {Z}^n_+)^{{{\,\textrm{Hom}\,}}(F,\mathbb {C})}.$ We say that a cuspidal automorphic representation $\pi$ is *regular algebraic*^9^*/cohomological of weight *$\lambda$ if $\pi _\infty$ has the same infinitesimal character as $M_{\lambda ,\mathbb {C}}^{\vee }$.

#### Lemma 2.8

Let *v* be an infinite place of *F*. Let $(\rho ,V)$ be an irreducible admissible representation of $G(F_v)$ that has central character $\omega$. Then the infinitesimal character of $\rho$ determines $\omega |_{F_v^{\times ,\circ }}$, where $F_v^{\times ,\circ }$ is the identity component of $F_v^\times$.

#### Proof

First assume *v* is a real place. Let us use the corresponding real embedding to identify $F_v$ with $\mathbb {R}$. Then there is $s\in \mathbb {C}$ such that $\omega (y)=y^s$ for all $y\in F_v^{\times ,\circ }=\mathbb {R}_{>0}.$ Let $$X=\begin{pmatrix} 1 &  \\ &  \ddots & \\ &  &  1 \end{pmatrix}$$ as an element in the centre of the complexified universal enveloping algebra of $G(F_v)$. By definition of the Lie algebra action, for all $u\in V_{sm}$,

$$\begin{aligned} X\cdot u = \frac{d}{dt}\Big |_{t=0}\rho (e^{Xt})u=\frac{d}{dt}\Big |_{t=0} e^{st}u=su. \end{aligned}$$

Thus, the infinitesimal character determines *s* and hence $\omega |_{F_v^{\times ,\circ }}$.

Now, assume *v* is a complex place. Let $\sigma _1,\sigma _2:F\rightarrow \mathbb {C}$ be the two complex embeddings corresponding to *v*. Use the same notation for the induced isomorphisms $F_v\xrightarrow {\sim }\mathbb {C}.$ Then there are $s_1,s_2\in \mathbb {C}$ with $s_1-s_2\in \mathbb {Z}$ such that $\omega (y)=\sigma _1(y)^{s_1}\sigma _2(y)^{s_2}$ for all $y\in F_v^{\times }\;(\cong \;\mathbb {C}^\times ).$ For $x\in F_v$, let $$X=\begin{pmatrix} x &  \\ &  \ddots & \\ &  &  x \end{pmatrix}$$ as an element in the centre of the complexified universal enveloping algebra of $G(F_v)$. By definition of the Lie algebra action, for all $u\in V_{sm}$,

$$\begin{aligned} X\cdot u = \frac{d}{dt}\Big |_{t=0}\rho (e^{Xt})u=\frac{d}{dt}\Big |_{t=0} e^{s_1\sigma _1(x)t+s_2\sigma _2(x)t}u=(s_1\sigma _1(x)+s_2\sigma _2(x))u. \end{aligned}$$

Taking $x=1$ and $x=\sigma _1^{-1}(i)$ gives us two equations that allows us to solve for $s_1,s_2$. Thus, the infinitesimal character determines $s_1,s_2$ and hence $\omega |_{F_v^{\times }}$. $\square$

#### Lemma 2.9

If $\pi$ is a regular algebraic automorphic representation of weight $\lambda$, then the restriction of its central character to $F_\infty ^{\times \circ }$ is the inverse of that of $M_{\lambda ,\mathbb {C}}$. In particular, $A_G$ acts trivially on $\pi _\infty \otimes M_\lambda$.

#### Proof

Let $$M_{\lambda _v}:={\left\{ \begin{array}{ll} M_{\lambda _{\tau },\mathbb {C}} & \text {if }v\text { is real}\\ M_{\lambda _{\tau },\mathbb {C}}\otimes _\mathbb {C}M_{\lambda _{\bar{\tau }},\mathbb {C}} & \text {if }v\text { is complex} \end{array}\right. }$$, where $\tau$ is an embedding $F\rightarrow \mathbb {C}$ whose associated place is *v*. Then the infinitesimal character of $\pi _v$ is the same as that of $M_{\lambda _v}^\vee$. We want to show that the restriction of the central character of $\pi _v$ to $F_v^{\times ,\circ }$ is that of $M_{\lambda _v}^\vee =M_{\lambda _v^\vee }$. This follows from Lemma 2.8. $\square$

#### Definition 2.10

We define

$$\begin{aligned} b_n:=r_1 \left\lfloor n^2/4\right\rfloor +r_2 n(n-1)/2,\; t_n:= r_1 \left\lfloor (n+1)^2/4\right\rfloor +r_2 n(n+1)/2-1. \end{aligned}$$

If *n* is clear, we may just write *b* and *t* instead.^10^

#### Definition 2.11

Let $\pi$ be a regular algebraic cuspidal automorphic representation of weight $\lambda$. Let . We say that $\epsilon$ is a *permissible signature* if *n* is even, or *n* is odd and $\epsilon _v$ is the central character of $\pi _v\otimes M_{\lambda _v}$ restricted to $\{\pm 1\}$ for all real places *v*.

#### Lemma 2.12

Let $\pi$ be a regular algebraic cuspidal automorphic representation of weight $\lambda$. Let $\epsilon$ be a permissible signature. Then for $i\in \{b_n,t_n\}$, the space

$$\begin{aligned} H^{i}(\mathfrak g, K_\infty ^0,\pi _\infty \otimes _\mathbb {C}M_{\lambda ,\mathbb {C}})[\epsilon ] \end{aligned}$$

is 1 dimensional, where $[\epsilon ]$ denotes the $\epsilon$-isotypic component.

The strategy is to use Künneth formula and Clozel’s result for Lie algebra cohomology of $\pi _v\otimes M_{\lambda _v}$. A slight complication is caused by the fact that our $K_\infty$ (which contains $A_G$) does not factors as a product over the infinite places.

#### Proof

Let

$$\begin{aligned} G^1(F_\infty )&:=\left\{ (g_v)\in G(F_\infty ): \prod _{v\mid \infty }|\det g_v|_{F_v}=1\right\} ,\\ G^1(F_v)&:=\{g_v\in G(F_v): |\det g_v|_v=1\},\\ H&:=\left\{ (a_v)\in \prod _{v\mid \infty }\mathbb {R}_{>0}:\prod _v |a_v|_{F_v}=1\right\} \subset G(F_\infty ), \end{aligned}$$

where we view $\prod _{v\mid \infty }\mathbb {R}_{>0}$ as a subgroup of the centre of $G(F_\infty )$ via the diagonal embedding. Let $\mathfrak g^1_\infty , \mathfrak g^1_v, \mathfrak h$ be the corresponding Lie algebras. Let

$$K^1_v:= {\left\{ \begin{array}{ll} O_n(\mathbb {R}) \text { if }v\text { is real} \\ U_n(\mathbb {R}) \text { if }v\text { is complex} \end{array}\right. }, $$

which is a subgroup of $G^1(F_v)$, so $\mathfrak k^1_v:=Lie(K^1_v) \subset \mathfrak g^1_v.$

From the Lie group decompositions $G(F_\infty )= G^1(F_\infty ) \times A_G$ and $K_\infty = \prod _{v\mid \infty }K^1_v \times A_G$, we get corresponding decompositions of Lie algebras, which in turns give

1 $$\begin{aligned} \mathfrak g/\mathfrak k_\infty = \mathfrak g^1_\infty /\prod _{v\mid \infty }\mathfrak k^1_v \end{aligned}$$

as $\mathbb {C}[K_\infty ]$-modules, where $K_\infty$ acts by conjugation.

Similarly, from the Lie group decomposition $G^1(F_\infty ) =\prod _{v\mid \infty }G^1(F_v) \times H$, we get a corresponding Lie algebra decomposition for $\mathfrak g^1_\infty$, which we can substitute to equation (1) to get

2 $$\begin{aligned} \mathfrak g/\mathfrak k_\infty = \left( \prod _{v\mid \infty }\frac{\mathfrak g^1_v}{\mathfrak k^1_v}\right) \times \mathfrak h \end{aligned}$$

as $\mathbb {C}[K_\infty ]$-modules. Note that the action of $K_\infty$ on $\mathfrak h$ is trivial.

The relative Lie algebra complex computing $H^{i}(\mathfrak g, K_\infty ^0,\pi _\infty \otimes _\mathbb {C}M_{\lambda ,\mathbb {C}})$ is by definition the *i*-th cohomology of

$$\begin{aligned}&\;\;(\wedge ^* (\mathfrak g/\mathfrak k_\infty )^\vee \otimes _\mathbb {R}(\pi _\infty )_{K_\infty \text {-fin}} \otimes _\mathbb {C}M_{\lambda ,\mathbb {C}})^{K_\infty ^\circ }\\ =&\left( \wedge ^* \left( \prod _{v\mid \infty }\frac{\mathfrak g^1_v}{\mathfrak k^1_v}\right) ^\vee \otimes _\mathbb {R}\wedge ^*\mathfrak h^\vee \otimes _\mathbb {R}(\pi _\infty )_{K_\infty \text {-fin}} \otimes _\mathbb {C}M_{\lambda ,\mathbb {C}}\right) ^{K_\infty ^\circ } \end{aligned}$$

where $(\pi _\infty )_{K\text {-fin}}$ means the $K_\infty$-finite vectors of $\pi _\infty$. As the action of $K_\infty$ on $\mathfrak h$ is trivial, we can pull out that factor from the cohomology and get

$$\begin{aligned} H^{i}(\mathfrak g, K_\infty ^0,\pi _\infty \otimes _\mathbb {C}M_{\lambda ,\mathbb {C}}) = \bigoplus _{a+b=i} H^a\left( \left( \wedge ^* \left( \prod _{v\mid \infty }\frac{\mathfrak g^1_v}{\mathfrak k^1_v}\right) ^\vee \otimes _\mathbb {R}(\pi _\infty )_{K_\infty \text {-fin}} \otimes _\mathbb {C}M_{\lambda ,\mathbb {C}}\right) ^{K_\infty ^\circ }\right) \otimes _\mathbb {R}\wedge ^b\mathfrak h^\vee . \end{aligned}$$

Since $A_G$ acts trivially on $\wedge ^* \left( \prod _{v\mid \infty }\frac{\mathfrak g^1_v}{\mathfrak k^1_v}\right) ^\vee$ and $\pi _\infty \otimes _\mathbb {C}M_{\lambda ,\mathbb {C}}$ by Lemma 2.9, we can replace $K_\infty ^\circ$ on the right hand side by $\prod _{v\mid \infty }K_v^1$. Then by definition

$$\begin{aligned} H^{i}(\mathfrak g, K_\infty ^0,\pi _\infty \otimes _\mathbb {C}M_{\lambda ,\mathbb {C}}) = \bigoplus _{a+b=i}H^{a}\left( \prod _{v\mid \infty }\mathfrak g^1_v, \prod _{v\mid \infty }\mathfrak k^1_v,\pi _\infty \otimes _\mathbb {C}M_{\lambda ,\mathbb {C}}\right) \otimes _\mathbb {R}\wedge ^b\mathfrak h^\vee , \end{aligned}$$

which by Künneth formula [16, section 1.3 equation (2)] equals

$$\begin{aligned} \bigoplus _{a_1+\dots +a_{m}+b=i}H^{a_1}\left( \mathfrak g^1_v,\mathfrak k^1_v,\pi _v\otimes _\mathbb {C}M_{\lambda _v,\mathbb {C}}\right) \otimes _\mathbb {C}\cdots \otimes _\mathbb {C}H^{a_m}\left( \mathfrak g^1_v,\mathfrak k^1_v,\pi _v\otimes _\mathbb {C}M_{\lambda _v,\mathbb {C}}\right) \otimes _\mathbb {R}\wedge ^b\mathfrak h^\vee , \end{aligned}$$

where $m:=r_1+r_2$, $$M_{\lambda _v,\mathbb {C}}:={\left\{ \begin{array}{ll} M_{\lambda _{\tau },\mathbb {C}} & \text {if }v\text { is real}\\ M_{\lambda _{\tau },\mathbb {C}}\otimes _\mathbb {C}M_{\lambda _{\bar{\tau }},\mathbb {C}} & \text {if }v\text { is complex} \end{array}\right. }$$, and $\tau$ is an embedding $F\rightarrow \mathbb {C}$ whose associated place is *v*. The result now follows from [9, Lemma 3.14], Remark 2.13, and the fact that $\mathfrak h\cong \mathbb {R}^{r_1+r_2-1}$. $\square$

#### Remark 2.13

For even *n*, [9, Lemma 3.14] only worked with trivial $\epsilon _v$. To deduce the result for general $\epsilon$, one can twist $\pi$ by a suitable Hecke character $F^\times \backslash \mathbb {A}_F^\times \rightarrow \{\pm 1\}$, which can for instance be constructed from a suitable character of $\overline{F^\times F_\infty ^{\times ,\circ }}\backslash \mathbb {A}_F^\times \cong {{\,\textrm{Gal}\,}}(F^{ab}/F)$.

### More on cohomology

#### Definition 2.14

We define the *cuspidal cohomology* as

$$\begin{aligned} H^*_{cusp}(X_U,M_{\mu ,\mathbb {C}}):=H^*(\mathfrak g,K_\infty ^\circ , M_{\mu ,\mathbb {C}}\otimes _\mathbb {C}L^2_0(G(F)\backslash G(\mathbb {A}_F),\chi )^U), \end{aligned}$$

where $\chi ^{-1}$ is the restriction to $A_G$ of the central character of $G(F\otimes \mathbb {C})$ on $M_{\mu ,\mathbb {C}}$.

The cuspidal cohomology is also acted on by $\mathcal {H}(G(\mathbb {A}_F^{\infty }),U)$ and there is a $\mathcal {H}(G(\mathbb {A}_F^{\infty }),U)$-equivariant injection

$$\begin{aligned} H^*_{cusp}(X_U,M_{\mu ,\mathbb {C}}) \hookrightarrow H^*(X_U,M_{\mu ,\mathbb {C}}). \end{aligned}$$

By multiplicity one and semisimplicity of $L^2_0(G(F)\backslash G(\mathbb {A}_F),\chi )$ [15, Corollary 9.1.3, Theorem 11.4.3], we know

$$\begin{aligned} L^2_0(G(F)\backslash G(\mathbb {A}_F),\chi )=\widehat{\bigoplus }_{\pi }\pi , \end{aligned}$$

where the sum ranges over all cuspidal automorphic representations of character $\chi$. We have a corresponding decomposition of the cuspidal cohomology into finite algebraic sum [8, Theorem 4.1], [9, Lemma 3.15]:

$$\begin{aligned} H^*_{cusp}(X_U,M_{\mu ,\mathbb {C}})= \oplus _\pi H^*(\mathfrak g,K_\infty ^\circ , M_{\mu ,\mathbb {C}}\otimes _\mathbb {C}\pi _\infty )\otimes _\mathbb {C}(\pi ^\infty )^U. \end{aligned}$$

By strong multiplicity one, for every cuspidal automorphic representation of character $\chi$, the $(\pi ^{\infty ,S})^{U^S}$-isotypic component is

3 $$\begin{aligned} H^*_{cusp}(X_U,M_{\mu ,\mathbb {C}})[(\pi ^{\infty ,S})^{U^S}]=H^*(\mathfrak g,K_\infty ^\circ ,\pi _\infty \otimes _\mathbb {C}M_{\mu ,\mathbb {C}})\otimes _\mathbb {C}(\pi ^\infty )^U. \end{aligned}$$

By Lemma 2.12, if $\pi$ is regular algebraic cuspidal of weight $\lambda$ with $\pi ^U\ne 0$, then there is a $\mathcal {H}(\textrm{GL}_n(\mathbb {A}_F^\infty ),U)$-equivariant injection $(\pi ^\infty )^U \hookrightarrow H^{b_n}_{cusp}(X_U,M_{\lambda ,\mathbb {C}})$. The same holds for the top degree $t_n$.

#### Definition 2.15

We define the *inner cohomology* by $H^*_!=im(H^*_c \rightarrow H^*)$, where $H^*_c$ is the compactly supported cohomology.

Then $H^*_!(X_U,M_{\mu ,A})$ for $A\in \{\mathcal {O},E,\mathbb {C}\}$ is also acted on by the Hecke algebras by restriction of the action on $H^*(X_U,M_{\mu ,A})$. Moreover, there is a Hecke-equivariant injection

$$\begin{aligned} H^*_{cusp}(X_U,M_{\mu ,\mathbb {C}}) \hookrightarrow H^*_!(X_U,M_{\mu ,\mathbb {C}}). \end{aligned}$$

#### Lemma 2.16

- $\mathcal {H}_\mathbb {C}:=\mathcal {H}(G^S,U^S)\otimes _\mathbb {Z}\mathbb {C}$ acts semisimply on $H^*_!(X_U,M_{\mu ,\mathbb {C}})$ and there is a decomposition
  $$\begin{aligned} H^*_!(X_U,M_{\mu ,\mathbb {C}})\cong \bigoplus _{\Pi \in \Pi _d} m(\Pi )(\Pi ^{\infty ,S})^{U^S}, \end{aligned}$$
  where $\Pi _d$ is the set of isomorphism classes of all discrete automorphic representations occurring as a subrepresentation of $L^2_d(\textrm{GL}_n(F)\backslash \textrm{GL}_n(\mathbb {A}_F),\chi )$ and $m(\Pi )\in \mathbb {Z}_{\ge 0}$.
- The $(\pi ^{\infty ,S})^{U^S}$-isotypic component is
  $$\begin{aligned} H^*_!(X_U,M_{\mu ,\mathbb {C}})[(\pi ^{\infty ,S})^{U^S}]=H^*(\mathfrak g,K_\infty ^\circ ,\pi _\infty \otimes _\mathbb {C}M_{\mu ,\mathbb {C}})\otimes _\mathbb {C}(\pi ^\infty )^U. \end{aligned}$$
- If $U^S:=\textrm{GL}_n(\prod _{v\notin S\cup \{\infty \}}\mathcal {O}_{F_v})$, then the ring
  $$\begin{aligned} \mathbb {T}^S_\mathbb {C}(H^*_!(X_U,M_{\mu ,\mathbb {C}})):=im(\mathcal {H}_\mathbb {C}\rightarrow {{\,\textrm{End}\,}}_\mathbb {C}(H^*_!(X_U,M_{\mu ,\mathbb {C}}))) \end{aligned}$$
  is reduced.

#### Proof

Let $H_2^*=H^*(\mathfrak g,K_\infty ^\circ ,L^2_d(\textrm{GL}_n(F)\backslash \textrm{GL}_n(\mathbb {A}_F),\chi )_{sm}\otimes _\mathbb {C}M_{\mu ,\mathbb {C}}),$ where $()_{sm}$ stands for the smooth vectors. We have a Hilbert space decomposition

$$\begin{aligned} L^2_d(\textrm{GL}_n(F)\backslash \textrm{GL}_n(\mathbb {A}_F),\chi )\cong \widehat{\bigoplus }_{\Pi \in \Pi _d}m_d(\Pi )\Pi . \end{aligned}$$

By the multiplicity one theorem for the discrete spectrum proved by Moeglin and Waldspurger [17], we have $m_d(\Pi )=1$ for all such $\Pi$. We have a corresponding decomposition for the cohomology [18, Theorem 5.3]:

4 $$\begin{aligned} H_2^*\cong \bigoplus _{\Pi \in \Pi _d} H^*(\mathfrak g,K_\infty ^\circ ,\Pi _\infty \otimes _\mathbb {C}M_{\mu ,\mathbb {C}})\otimes _\mathbb {C}(\Pi ^\infty )^U. \end{aligned}$$

Each $\Pi ^\infty$ is an irreducible admissible representation of $\textrm{GL}_n(\mathbb {A}_F^\infty )$, so it is factorisable, so $(\Pi ^\infty )^U=(\Pi _S)^{U_S}\otimes _\mathbb {C}(\Pi ^{\infty ,S})^{U^S}$ is isomorphic to a direct sum of simple modules of $\mathcal {H}_\mathbb {C}$. It follows that $H_2^*$ is a semisimple $\mathcal {H}_\mathbb {C}$-module.

By a result of Borel [9, Proposition 3.18], we have injections

5 $$\begin{aligned} H^*_{cusp}(X_U,M_{\mu ,\mathbb {C}}) \hookrightarrow H^*_{!}(X_U,M_{\mu ,\mathbb {C}}) \hookrightarrow \tilde{H}^*_2 \end{aligned}$$

where $\tilde{H}^*_2$ is the image of $H^*_2\rightarrow H^*(X_U,M_{\mu ,\mathbb {C}}).$ Submodules of semisimple modules are semisimple, so $H^*_{!}(X_U,M_{\mu ,\mathbb {C}})$ is a semisimple $\mathcal {H}_\mathbb {C}$-module and part (a) follows.

For part (b), recall that discrete automorphic representations are isobaric (as they are Speh representations), so they satisfy strong multiplicity one, so $H_2^*[(\pi ^{\infty ,S})^{U^S}]=H^*(\mathfrak g,K_\infty ^\circ ,\pi _\infty \otimes _\mathbb {C}M_{\mu ,\mathbb {C}})\otimes _\mathbb {C}(\pi ^\infty )^U$ by (4). But this is also the $(\pi ^{\infty ,S})^{U^S}$-isotypic component of the cuspidal cohomology by equation (3). Part (b) now follows from (5).

For part (c), assume $U^S:=\textrm{GL}_n(\prod _{v\notin S\cup \{\infty \}}\mathcal {O}_{F_v})$. By Satake isomorphism, $\mathcal {H}_\mathbb {C}$ is commutative. Thus, $(\Pi ^{\infty ,S})^{U^S}$ is 0-dimensional or 1-dimensional. Hence each element of $\mathcal {H}_\mathbb {C}$ acts on $(\Pi ^{\infty ,S})^{U^S}$ by a scalar. The result now follows from part (a). $\square$

## Abstract congruence ideals

In this section, we will define congruence ideals as in [4, section 2.1]. We will also establish some of their properties in the abstract algebraic setting. These will be applied to the Hecke algebras and cohomology of locally symmetric space in the next section.

Let $\mathcal {O}$ be a complete discrete valuation ring with uniformizer $\varpi$ and field of fractions *E*. Let *T* be a reduced finite flat local $\mathcal {O}$-algebra, $\lambda :T\rightarrow \mathcal {O}$ be an $\mathcal {O}$-algebra homomorphism. This, being a section of the structure map $\mathcal {O}\rightarrow T$, is necessarily surjective. We first recall a standard concept, which already appeared in [3]:

### Definition 3.1

$$\eta _\lambda :=\lambda (Ann_T (\ker \lambda ))$$.

It turns out that to study $\eta _\lambda$, it is useful to generalise this concept to modules over *T*.

Let *M* be a finitely generated *T*-module which is free over $\mathcal {O}$. Write $M_E=M\otimes _{\mathcal {O}}E$ and $T_E=T\otimes _{\mathcal {O}}E$ Note that $M\hookrightarrow M_E$ and $T\hookrightarrow T_E$ by $\mathcal {O}$-flatness of *T* and *M*. Also, $\lambda$ induces an *E*-algebra map $\lambda _E:T_E\rightarrow E$. Note that $T_E$ is a finite dimensional *E*-vector space, so it is Artinian. It follows that

6 $$\begin{aligned} T_E&\cong \prod _{\mathfrak p\in Spec T_E} (T_E)_{\mathfrak p} \end{aligned}$$

7 $$\begin{aligned}&\cong \prod _{\mathfrak p\in Spec T_E} T_E/\mathfrak p\end{aligned}$$

8 $$\begin{aligned}&\cong E\times \prod _{\mathfrak p\ne ker \lambda _E} T_E/\mathfrak p \end{aligned}$$

Here, (6) is given by the diagonal map; (7) is true as $T_E$ is reduced; (8) is true by the first isomorphism theorem. The upshot is that we have a canonical decomposition

$$\begin{aligned} T_E\cong E\times T_E^c \end{aligned}$$

of *E*-algebras, where the first projection is given by $\lambda _E$.

Let $e_\lambda =e\in T_E$ be the element corresponding to $(1,0)\in E\times T_E^c$. In other words, *e* is the unique element of $T_E$ such that $\lambda _E(e)=1$ and $e\in \bigcap _{\mathfrak p\ne \ker \lambda _E}\mathfrak p$. Define two *T*-submodules of $M_E$ by

$$\begin{aligned} M^\lambda :=eM \end{aligned}$$

and

$$\begin{aligned} M_\lambda :=eM\cap M = M[\ker \lambda ] \end{aligned}$$

where the last equality is proved in Lemma 3.3. In [4, section 2.1], $M_\lambda$ is defined as $eM_E\cap M$, but it is equivalent to our definition, because if $m\in eM_E\cap M$, then $m=em\in M\cap eM$.

### Definition 3.2

Define the *congruence module* $C_0^\lambda (M)$ by

$$\begin{aligned} C_0^\lambda (M):=\frac{M^\lambda }{M_\lambda } \end{aligned}$$

and the *congruence ideal* to be its Fitting ideal

$$\begin{aligned} \eta _\lambda (M):=Fitt_{\mathcal {O}}(C_0^\lambda (M)). \end{aligned}$$

Note that $C_0^\lambda (M)$ is a finite torsion $\mathcal {O}$-module, so $\eta ^\lambda (M)$ is completely determined by the cardinality of $C_0^\lambda (M)$. More precisely, if $C_0^\lambda (M)$ has cardinality $|\mathcal {O}/\varpi |^a$, then $\eta _\lambda (M)=(\varpi ^a)$.

To see how definitions 3.1, 3.2 are related, note:

### Lemma 3.3

Let *M* and *T* be as above.

1. $$M_\lambda =M[\ker \lambda ]:=\{m\in M:(\ker \lambda ) m=0\}$$.
2. $$\eta _\lambda (T)=\eta _\lambda $$.

### Proof

For (1), we first consider a slightly more general setup. Suppose we have a product of commutative rings $A\times B$ acting on *N*. We can decompose *N* into $N_1\times N_2$ accordingly. It is then clear that $N_1=N[\ker \pi _A]$, where $\pi _A:A\times B\rightarrow A$ is the first projection.

In our case, taking $A\times B=E\times T_E^c$ and $N=M_E$ shows $e M_E=M_E[\ker \lambda _E]$. Hence $M_\lambda =e M_E \cap M=\{m\in M: m\otimes 1\in M_E \text { is annihilated by }(\ker \lambda )\otimes _\mathcal {O}E\}=M[\ker \lambda ]$ since *M* is a free $\mathcal {O}$-module.

For (2), note that under the $\mathcal {O}$-module isomorphism $T_E\cong E\times T^c_E$, the $\mathcal {O}$-module $T^\lambda =eT$ corresponds to $\lambda (T)\times 0=\mathcal {O}\times 0$ while $T_\lambda =T[\ker \lambda ]$ corresponds to $\lambda (T[\ker \lambda ])\times 0$. Hence, $C_0^\lambda (T)=\mathcal {O}/\lambda (T[\ker \lambda ])$ and $\eta _\lambda (M)=\lambda (T[\ker \lambda ])=\eta _\lambda$. $\square$

Observe that $M^\lambda =eM \subset M_E$ is torsion free and finitely generated over $\mathcal {O}$, so it is a free $\mathcal {O}$-module. The same is true for $M_\lambda =M[\ker \lambda ]$.

### Lemma 3.4

(cf. [4, equation (2.2)]) If $rk_\mathcal {O}M_\lambda =1$, then $C_0^\lambda (M)=\mathcal {O}/\eta _\lambda (M)$ and $\eta _\lambda (M)\supset \eta _\lambda$.

### Proof

We know that $M^\lambda /M_\lambda$ is a finite torsion $\mathcal {O}$-module, so $rk_\mathcal {O}(M^\lambda )=rk_\mathcal {O}(M_\lambda )=1$. Thus, there exists $m\in M$ such that $eM=\mathcal {O}em$ as $\mathcal {O}$-module. Then we have a surjection of $\mathcal {O}$-modules

$$\begin{aligned} C_0^\lambda (T)=\frac{eT}{T[\ker \lambda ]}&\twoheadrightarrow C_0^\lambda (M)=\frac{eM}{M[\ker \lambda ]}\\ x&\mapsto xm. \end{aligned}$$

The lemma now follows from the observation that $C_0^\lambda (T)=\mathcal {O}/ \eta _\lambda$. $\square$

### Lemma 3.5

Let $T,\lambda , e$ be as above. Let $\tilde{T}$ be a finite flat local $\mathcal {O}$-algebra, $\tilde{\lambda }:\tilde{T}\rightarrow \mathcal {O}$ be an $\mathcal {O}$-algebra homomorphism, and $\tilde{e}$ be the corresponding idempotent in $\tilde{T}$. Let $M_1,M_2$ be *T*-module and $\tilde{T}$-module respectively that are finite free over $\mathcal {O}$. Suppose that there is an $\mathcal {O}$-bilinear perfect pairing^11^

$$\begin{aligned} {[}\,,\,]:M_1\times M_2 \rightarrow \mathcal {O}\end{aligned}$$

such that^12^$[eM_1,(1-\tilde{e})M_2]=0$ and $[(1-e)M_1,\tilde{e}M_2]=0$.

- Then $[,\,]$ induces an $\mathcal {O}$-bilinear perfect pairing
  $$\begin{aligned} C_0^\lambda (M_1)\times C_0^{\tilde{\lambda }}(M_2) \rightarrow E/\mathcal {O}\end{aligned}$$
  and $$\eta _\lambda (M_1)=\eta _{\tilde{\lambda }}(M_2)$$.
- If $M_1[\ker \lambda ]$ and $M_2[\ker \tilde{\lambda }]$ are both free $\mathcal {O}$-modules of rank 1 with respective bases $\delta _1,\delta _2$, then
  $$\begin{aligned} \eta _\lambda (M_1)=\eta _{\tilde{\lambda }}(M_2)=([\delta _1,\delta _2]). \end{aligned}$$

The key observation is that for every finite torsion $\mathcal {O}$-module *N*, we have a (non-canonical) isomorphism $N\cong {{\,\textrm{Hom}\,}}_\mathcal {O}(N,E/\mathcal {O})$. Hence, by considering the cardinalities of $C_0^\lambda (M_1)$ and $C_0^{\tilde{\lambda }}(M_2)$, we know the pairing in (a) is perfect iff it is non-degenerate.

This lemma appears similar to [4, Proposition 2.3], but a key difference is that we do not assume $[tx,y]=[x,ty]$ for all $t\in T$. Instead, our analogous conditions are $[eM_1,(1-\tilde{e})M_2]=0$ and $[(1-e)M_1,\tilde{e}M_2]=0$. This distinction is important because we will later apply this lemma to the cup product, which satisfies our conditions but not theirs. In the $\textrm{GL}_2$ setting, they twisted the pairing by the Atkin-Lehner involution to make their conditions hold, but we are unaware of any such involution for $\textrm{GL}_n$.

### Proof

It is easy to see that $[,\,]$ extends to an *E*-bilinear perfect pairing $[,\,]:(M_1)_E\times (M_2)_E \rightarrow E$. Let $em_1\in eM_1$ (with $m_1\in M_1$) and $\tilde{e}m_2\in M_2\cap \tilde{e}M_2$ (with $m_2\in M_2$). Then $[em_1,\tilde{e}m_2]=[em_1+(1-e)m_1,\tilde{e}m_2]=[m_1,\tilde{e}m_2]\in \mathcal {O}$ because $(m_1,\tilde{e}m_2)\in M_1\times M_2$. A symmetric consideration shows $[,\,]$ induces a map

$$\begin{aligned} \langle \,,\,\rangle :C_0^\lambda (M_1)\times C_0^{\tilde{\lambda }}(M_2) \rightarrow E/\mathcal {O}. \end{aligned}$$

To show $\langle ,\,\rangle$ is non-degenerate, we let $m_1\in M_1$. Suppose that $\langle em_1,-\rangle \in {{\,\textrm{Hom}\,}}_\mathcal {O}(C_0^{\tilde{\lambda }}(M_2),E/\mathcal {O})$ is zero. This means that for all $m_2\in M_2$,

$$\begin{aligned} {[}em_1,\tilde{e}m_2]=[em_1,\tilde{e}m_2+(1-\tilde{e})m_2]=[em_1,m_2]\in \mathcal {O}\end{aligned}$$

so $[em_1,-]\in {{\,\textrm{Hom}\,}}_\mathcal {O}(M_2,\mathcal {O})$. By perfectness, there exists $n\in M_1$ such that $[em_1,-]=[n,-]$, so $n=em_1\in eM_1\cap M_1=(M_1)_\lambda$ by perfectness again, so $em_1=0\in C_0^\lambda (M_1)$. By symmetry, $\langle \,, \, \rangle$ is non-degenerate and hence perfect. Also, $C_0^\lambda (M_1)\cong {{\,\textrm{Hom}\,}}_\mathcal {O}(C_0^{\tilde{\lambda }}(M_2),E/\mathcal {O})\cong C_0^{\tilde{\lambda }}(M_2).$

For part (b), let $(\varpi ^a):=\eta _\lambda (M_1)=\eta _{\tilde{\lambda }}(M_2)$. By Lemma 3.4, $C_0^\lambda (M_1)$ and $C_0^{\tilde{\lambda }}(M_2)$ are free $\mathcal {O}/(\varpi ^a)$-modules of rank 1, with respective bases $b_1,b_2$ say. By part (a), we have isomorphism

$$\begin{aligned} \frac{\mathcal {O}}{(\varpi ^a)} \cong \frac{\mathcal {O}}{(\varpi ^a)}b_1\xrightarrow {\sim } {{\,\textrm{Hom}\,}}_\mathcal {O}\left( \frac{\mathcal {O}}{(\varpi ^a)}b_2,E/\mathcal {O}\right)&\xrightarrow {\sim }\frac{\varpi ^{-a}\mathcal {O}}{\mathcal {O}}\xrightarrow {\cdot \varpi ^a}\frac{\mathcal {O}}{(\varpi ^a)}\\ f&\mapsto f(b_2). \end{aligned}$$

This isomorphism maps 1 to $[b_1,b_2]\varpi ^a \pmod {\varpi ^a}$, so $[b_1,b_2]\varpi ^a\in \mathcal {O}^\times$. As $\mathcal {O}b_i/\mathcal {O}\delta _i \cong \mathcal {O}/(\varpi ^a)$, we know $\delta _i\in \varpi ^a b_i\mathcal {O}^\times$. We deduce that $[\delta _1,\delta _2]\in \varpi ^a\mathcal {O}^\times$, as desired. $\square$

The following lemma explains why $\eta _\lambda$ is called the congruence ideal:

### Lemma 3.6

Let *E* be a non-Archimedean local field with ring of integers $\mathcal {O}$, *T* be a reduced finite flat local $\mathcal {O}$-algebra, $\lambda :T\rightarrow \mathcal {O}$ be an $\mathcal {O}$-algebra homomorphism. Then $\eta _\lambda \ne \mathcal {O}$ iff there is a finite field extension *L* of *E* and an $\mathcal {O}$-algebra homomorphism $\lambda ':T\rightarrow \mathcal {O}_{L}$ such that (viewing $\lambda$ as a homomorphism to $\mathcal {O}_L$) we have $\lambda \ne \lambda '$ and

$$\begin{aligned} \lambda \equiv \lambda ' \pmod {\varpi _L}. \end{aligned}$$

Here $\mathcal {O}_L$ is the ring of integers of *L* and $\varpi _L$ is a uniformizer of $\mathcal {O}_L$.

### Remark 3.7

Recall that *T* is finite over $\mathcal {O}$, so any $\mathcal {O}$-algebra homomorphism $T\rightarrow \overline{E}$ has image in $\mathcal {O}_L$ for some finite field extension *L* of *E*. Thus, we can rephrase the lemma: $\eta _\lambda \ne \mathcal {O}$ iff there is an $\overline{E}$-algebra homomorphism $\lambda ':T\otimes _{\mathcal {O}}\overline{E} \rightarrow \overline{E}$ such that $\lambda '\ne \lambda \otimes _{\mathcal {O}}\overline{E}$ and $|\lambda (t)-\lambda '(t)|<1$ for all $t\in T$, where $|\cdot |$ is an absolute value on $\overline{E}$ extending that of *E*.

### Proof

Given $\lambda$, we can decompose $T\otimes _\mathcal {O}E\cong E\times T_E^c$ and get an idempotent $e\in T\otimes _\mathcal {O}E$ as before. Define $T^c:=im(T\rightarrow T\otimes _\mathcal {O}E\rightarrow T_E^c).$

We assume that $\lambda '$ is as in the statement of the lemma. We claim that $\lambda '$ factors through $T\rightarrow T^c$. As $\mathcal {O}_L\subset \mathcal {O}_L\otimes _\mathcal {O}E$, it suffices to show

$$\begin{aligned} \lambda '_E:=\lambda '\otimes E: T\otimes _\mathcal {O}E\rightarrow \mathcal {O}_L\otimes _\mathcal {O}E \end{aligned}$$

factors through $T^c_E$. Note that $\mathcal {O}_L\otimes _\mathcal {O}E=L$ is an integral domain while $T\otimes _\mathcal {O}E\cong E\times T_E^c$, so $\lambda '_E$ must factor through *E* or $T_E^c$. Since $\lambda '_E$ is an *E*-algebra homomorphism and $\lambda '_E \ne \lambda _E$, $\lambda '_E$ cannot factor through *E*. Hence $\lambda '_E$ must factor through $T_E^c$, as claimed. Suppose $\eta _\lambda =\mathcal {O}$. This means $eT\cap T = eT$, i.e. $T\supset eT$. In particular, $(1,0)=e\cdot (1,1)\in T$. Then

$$\begin{aligned} 1&=\lambda (1,0)\\&\equiv \lambda '(1,0)\pmod {\varpi _L}\\&\equiv 0\pmod \varpi \end{aligned}$$

where the last equality holds since $\lambda '$ factors through $T^c$. We get a contradiction, so $\eta _\lambda \ne \mathcal {O}$.

Conversely, suppose $\eta _\lambda \ne \mathcal {O}$. The key observation is that $\mathcal {O}/\eta _\lambda =\mathcal {O}\otimes _T T^c$, because $\ker (T\twoheadrightarrow T^c)=T\cap eT$. Thus we have an $\mathcal {O}$-algebra homomorphism

$$\begin{aligned} f:T^c\twoheadrightarrow \mathcal {O}\otimes _T T^c=\mathcal {O}/\eta _\lambda \twoheadrightarrow \mathcal {O}/\varpi . \end{aligned}$$

We get a commutative diagram 
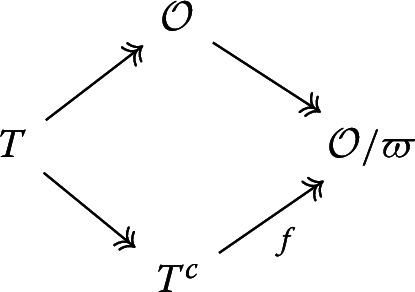
 We want to lift *f*. There is a classical argument due to Deligne and Serre. Let $\mathfrak {m}^c:=\ker f$. Consider the structure map $\mathcal {O}\rightarrow T^c$. As $\varpi \in \mathfrak {m}^c$, the prime ideal $\mathfrak {m}^c$ lies above $\varpi \mathcal {O}$. Note that multiplication by $\varpi$ is invertible in $T_E$, so $T_E$ and hence $T^c$ is $\mathcal {O}$-torsion free. Thus, $T^c$ is a finite free $\mathcal {O}$-module. By flatness, the going down property holds, so there is a prime ideal $\mathfrak p^c\subset \mathfrak {m}^c$ lying above (0), so $\mathcal {O}\hookrightarrow T^c/\mathfrak p^c$. This is a finite extension, so

$$\begin{aligned} L:= Frac(T^c/\mathfrak p^c) \end{aligned}$$

is a finite extension of *E*. We know $T^c/\mathfrak p^c$ is finite over $\mathcal {O}$ and hence integral over $\mathcal {O}$, so

$$\begin{aligned} T^c/\mathfrak p^c\subset \mathcal {O}_L. \end{aligned}$$

We want to show that this inclusion is a local homomorphism of local rings. As $\mathcal {O}$ is a Henselian ring and $T^c/\mathfrak p^c$ is an integral domain, $T^c/\mathfrak p^c$ is a local ring. Note $\mathfrak {m}':=\varpi _L\mathcal {O}_L \cap T^c/\mathfrak p^c$ is a prime ideal of $T^c/\mathfrak p^c$ lying above $\varpi \mathcal {O}$ (since its pullback to $\mathcal {O}$ is the preimage of $\varpi _L \mathcal {O}_L$ under the $\mathcal {O}$-algebra map $\mathcal {O}\rightarrow T^c/\mathfrak p^c \rightarrow \mathcal {O}_L$). By the incomparability theorem for injective integral ring extensions, $\mathfrak {m}'$ is a maximal ideal of $T^c/\mathfrak p^c$. Hence, the fact that $T^c/\mathfrak p^c$ is a local ring implies $\mathfrak {m}'=\mathfrak {m}^c/\mathfrak p^c$. This gives us another commutative diagram 
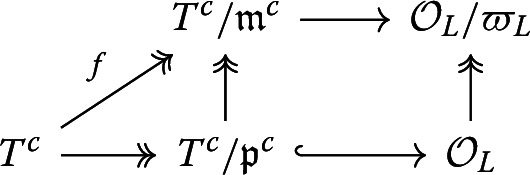
 Combining the two diagrams gives us 
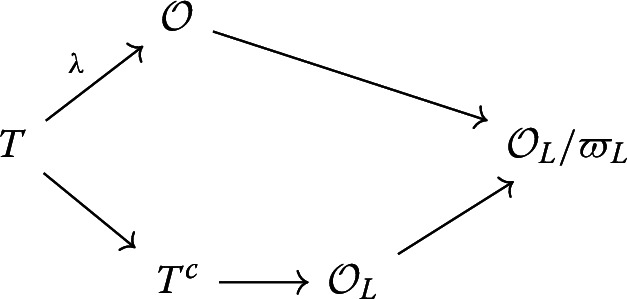
 Denote the bottom map $T\rightarrow \mathcal {O}_L$ by $\lambda '$. It remains to show $\lambda \ne \lambda '$ as maps to $\mathcal {O}_L$. If this is false, then for all $(x,y)\in T\subset \mathcal {O}\times T^c$, we have $x-y\in \mathfrak p^c$ by construction of $\lambda '$. In particular, if $(x,y)\in T\cap eT$, then $y=0$ and $x\in \mathcal {O}\cap \mathfrak p^c=(0)$ as $\mathfrak p^c$ lies above (0). This means $T\cap eT=0$, which is false as $\varpi ^a e\in T\cap eT$ for $a\in \mathbb {Z}$ sufficiently large. $\square$

## $L(1,\pi ,{{\,\textrm{Ad}\,}}^\circ )$ and congruences for automorphic representations

In this section, we will apply the results from the last section to study congruence ideals of Hecke algebras and cohomology of locally symmetric spaces. Then we will relate them to Selmer groups.

We shall need some notation. Let

- *F* be a number field
- *p* be a prime. Starting from section 4.2, we will also assume $p>2$.
- $\iota :\overline{\mathbb {Q}}_p\xrightarrow {\sim } \mathbb {C}$ be a fixed isomorphism
- $\pi$ be a cuspidal, regular algebraic automorphic representation of $\textrm{GL}_n(\mathbb {A}_F)$ of weight $\iota \mu$, where $\mu \in (\mathbb {Z}^n)^{{{\,\textrm{Hom}\,}}(F,\overline{\mathbb {Q}_p})}$
- $U=\prod _{v\not \mid \infty }U_v\subset \prod _{v\not \mid \infty }\textrm{GL}_n(\mathcal {O}_{F_v})$ be a neat open compact subgroup with $\pi ^{U}\ne 0$. Starting from section 4.2, we will also assume $(\pi ^\infty )^U$ is one dimensional^13^
- *S* be a finite set of finite places of *F* containing all *v* such that $\pi _v$ is ramified or $U_v\ne \textrm{GL}_n(\mathcal {O}_{F_v})$
- $$G^S=\textrm{GL}_n(\mathbb {A}_F^{S,\infty })$$ and $$U^S:=\textrm{GL}_n(\prod _{v\notin S\cup \{\infty \}}\mathcal {O}_{F_v})$$.
- $E\subset \overline{\mathbb {Q}}_p$ be a local field containing $\iota ^{-1}(\mathbb {Q}(\pi ))$^14^and the image of every embedding $F\hookrightarrow \overline{\mathbb {Q}}_p$
- $\mathcal {O}$ be the ring of integers of *E*, $\varpi$ a uniformizer of $\mathcal {O}$
- $\epsilon \in \widehat{K_\infty /K_\infty ^\circ }$ a permissible signature (Definition 2.11)
- $X_U$ the locally symmetric space of $\textrm{GL}_{n,F}$ (Section 2.1), $\partial X_U$ the boundary of its Borel-Serre compactification.
- $$\mathbb {T}^S:= \mathcal {H}(G^S,U^S)\otimes _\mathbb {Z}\mathcal {O}.$$

Note that $\mathbb {T}^S$ is a commutative $\mathcal {O}$-algebra. If *M* is an $\mathcal {O}$-module equipped with an $\mathcal {O}$-algebra homomorphism $\mathbb {T}^S\rightarrow {{\,\textrm{End}\,}}_\mathcal {O}(M)$, then we define

$$\begin{aligned} \mathbb {T}^S(M):=im(\mathbb {T}^S\rightarrow {{\,\textrm{End}\,}}_\mathcal {O}(M)). \end{aligned}$$

For completeness, let us also remark that in Lemma 4.1 below, we shall show that there is a Hecke eigensystem $\Lambda :\mathbb {T}^S\rightarrow \mathcal {O}$ attached to $\pi$. We will let

$$\begin{aligned} \mathfrak {m}:=\ker (\Lambda \pmod \varpi ). \end{aligned}$$

### Hecke eigensystems

We first show that we have a Hecke eigensystem attached to $\pi$.

#### Lemma 4.1

We have an $\mathcal {O}$-algebra homomorphism

$$\begin{aligned} \Lambda :\mathbb {T}^S\rightarrow \mathcal {O}\end{aligned}$$

sending $t\in \mathbb {T}^S$ to its eigenvalue on $(\iota ^{-1}\pi ^{\infty })^{U}$. This homomorphism factors through $\mathbb {T}^S(H^*_!(X_U,M_{\mu ,\mathcal {O}})),$ where $H^*_!=im(H_c^*\rightarrow H^*)$ is the inner cohomology and $H^*=\oplus _{i\ge 0}H^i$.

#### Proof

In this proof, we may sometime abuse notation and regard a $\mathbb {C}$-vector space as an $\mathcal {O}$-module via the map $\iota :\mathbb {Q}_p\xrightarrow {\sim } \mathbb {C}$. Let $\mathcal {H}:=\mathcal {H}(G^S,U^S)$.

As $\textrm{GL}_n(\mathbb {A}_F^S)$ acts on the $\overline{\mathbb {Q}}_p$-vector space $\iota ^{-1}\pi ^\infty$, we know $\mathbb {T}^S$ acts on the $U^S$-invariant $(\iota ^{-1}\pi ^\infty )^{U^S}$ and hence also on $(\iota ^{-1}\pi ^\infty )^{U}$. Moreover, for all finite $v\notin S$, $\pi _v$ is unramified so $\pi _v^{U_v}$ is a one dimensional $\overline{\mathbb {Q}}_p$-vector space. It follows that each element of $\mathbb {T}^S$ acts by a scalar on $(\iota ^{-1}\pi ^\infty )^{U}$, so we get an $\mathcal {O}$-algebra homomorphism

9 $$\begin{aligned} \mathbb {T}^S \rightarrow \overline{\mathbb {Q}}_p \end{aligned}$$

sending an element to its eigenvalue.

Note that $\mathbb {T}^S(H^*_!(X_U,M_{\mu ,\mathcal {O}}))$ acts on $H^*_!(X_U,M_{\mu ,\mathcal {O}})\otimes _{\mathcal {O}}\mathbb {C}\cong H^*_!(X_U,M_{\mu ,\mathbb {C}})$. Since $\pi$ is cohomological of weight $\mu$, we have $\mathcal {H}$-equivariant injections

$$\begin{aligned} (\pi ^\infty )^U \hookrightarrow H^*_{cusp}(X_U,M_{\mu ,\mathbb {C}})\hookrightarrow H^*_!(X_U,M_{\mu ,\mathbb {C}}). \end{aligned}$$

Pick any non-zero $x\in (\pi ^\infty )^U$ and let *y* be its image under this injection. For all $t\in \mathbb {T}^U$, its eigenvalue on $(\iota ^{-1}\pi ^\infty )^U$ only depends on how it acts on *y* and this is determined by the image of *t* in $\mathbb {T}^S(H^*_!(X_U,M_{\mu ,\mathcal {O}}))$. It follows that (9) factors through $\mathbb {T}^S(H^*_!(X_U,M_{\mu ,\mathcal {O}})).$ It remains to show that the image of (9) lies in $\mathcal {O}$.

We first show that its image lies in *E*. By [9, Proposition 3.1], there is a $\textrm{GL}_n(\mathbb {A}_F^\infty )$-stable $\mathbb {Q}(\pi )$-vector subspace *W* of $\pi ^\infty$ such that $\pi ^\infty =W\otimes _{\mathbb {Q}(\pi )}\mathbb {C}.$ Then $(\pi ^\infty )^U=W^U\otimes _{\mathbb {Q}(\pi )}\mathbb {C}.$ Let $h\in \mathcal {H}$. We have already seen that it acts by a scalar on $(\pi ^\infty )^U$, so the same is true for $W^U$. As $W^U$ is a $\mathbb {Q}(\pi )$-vector space, the scalar must lies in $\mathbb {Q}(\pi )$. Hence the image of (9) lies in $\iota ^{-1}(\mathbb {Q}(\pi ))\subset E$.

Finally, by the existence of Borel-Serre compactification of $X_U$, we know that $H^*_!(X_U,M_{\mu ,\mathcal {O}})$ is a finite $\mathcal {O}$-module, so

$$\begin{aligned} H^*_!(X_U,M_{\mu ,\mathcal {O}})/\mathcal {O}\text {-torsion} \end{aligned}$$

is a finite free $\mathcal {O}$-module stable under $\mathcal {H}$. Pick an $\mathcal {O}$-basis $\mathcal {B}$ for this module. We can then express the action of $\mathcal {H}$ on $H^*_!(X_U,M_{\mu ,\mathcal {O}})/\mathcal {O}\text {-torsion}$ by matrices with entries in $\mathcal {O}$. We can view $\mathcal {B}$ as a $\mathbb {C}$-basis for $H^*_!(X_U,M_{\mu ,\mathbb {C}})$. Then the action of $\mathcal {H}$ on $H^*_!(X_U,M_{\mu ,\mathbb {C}})$ is given by the same matrices. Hence each eigenvalue of $\mathcal {H}$ on this space is a root of a monic polynomial over $\mathcal {O}$. We know they lie in *E* by the previous paragraph, so they lie in *E* as $\mathcal {O}$ is integrally closed. $\square$

We let $\mathfrak m=\ker (\Lambda \mod \varpi )\subset \mathbb {T}^S$, which is a maximal ideal. We define

$$\begin{aligned} T:=\mathbb {T}^S(H^*_!(X_U,M_{\mu ,\mathcal {O}}))_\mathfrak {m}/\mathcal {O}\text {-torsion}. \end{aligned}$$

#### Lemma 4.2

*T* is a reduced finite flat^15^ local $\mathcal {O}$-algebra. Also, $\Lambda$ induces a local $\mathcal {O}$-algebra map

$$\begin{aligned} \lambda :T\rightarrow \mathcal {O}. \end{aligned}$$

#### Proof

Let $H:=H^*_!(X_U,M_{\mu ,\mathcal {O}})$. By the existence of Borel-Serre compactification of $X_U$, *H* is a finite $\mathcal {O}$-module, so $\mathbb {T}^S(H)$ is a finite $\mathcal {O}$-algebra. We let

$$\begin{aligned} \Lambda ':\mathbb {T}^S(H) \rightarrow \mathcal {O}\end{aligned}$$

be the map induced by $\Lambda$, which exists by Lemma 4.1, and let $q:\mathbb {T}^S\rightarrow \mathbb {T}^S(H)$ be the quotient map, so $\Lambda =\Lambda '\circ q$. Then $\mathfrak {m}\supset \ker q$, so $q(\mathfrak {m})$ is a maximal ideal of $\mathbb {T}^S(H)$ and hence

$$\begin{aligned} \mathbb {T}^S(H)_\mathfrak {m}= \mathbb {T}^S(H)_{q(\mathfrak {m})} \end{aligned}$$

is a finite local $\mathcal {O}$-algebra. The same is thus true for *T*. As *T* is a finitely generated torsion-free $\mathcal {O}$-module and $\mathcal {O}$ is a PID, *T* is flat over $\mathcal {O}$. Hence $T\hookrightarrow T\otimes _\mathcal {O}\mathbb {C}$. As $\mathbb {C}$ is $\mathcal {O}$-flat, $T\otimes _\mathcal {O}\mathbb {C}=\mathbb {T}^S_\mathbb {C}(H^*_!(X_U,M_{\mu ,\mathbb {C}}))_\mathfrak {m},$ which is reduced by Lemma 2.16. Thus, *T*, which injects into $T\otimes _\mathcal {O}\mathbb {C}$, is also reduced.

Note that $q(m)=\ker (\Lambda '\mod \varpi )$. It follows that $\Lambda '$ induces an $\mathcal {O}$-algebra map $\mathbb {T}^S(H^*_!(X_U,M_{\mu ,\mathcal {O}}))_{q(m)}\rightarrow \mathcal {O}$ and hence an $\mathcal {O}$-algebra map $\lambda :T\rightarrow \mathcal {O}$, i.e. a commutative diagram 
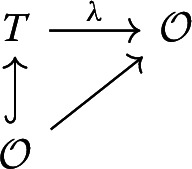
 It follows that $\lambda ^{-1}(\varpi \mathcal {O})$ is a prime ideal of *T* lying above $\varpi \mathcal {O}$ (i.e. the preimage of $\lambda ^{-1}(\varpi \mathcal {O})$ along the structure map $\mathcal {O}\rightarrow T$ is $\varpi \mathcal {O}$). Since *T* is a local $\mathcal {O}$-algebra, the maximal ideal of *T* also lies above $\varpi \mathcal {O}$. By the incomparability theorem for injective integral ring extensions, we deduce that $\lambda ^{-1}(\varpi \mathcal {O})$ is the maximal ideal of *T*. Thus, $\lambda$ is a local homomorphism. $\square$

#### Remark 4.3

Let us show that

$$\begin{aligned} T\cong \mathbb {T}^S(\overline{H}^*_!(X_U,M_\mu )_\mathfrak {m}), \end{aligned}$$

where $\overline{H}^*_!(X_U,M_\mu ):=H^*_!(X_U,M_\mu )/\mathcal {O}\text {-torsion}.$ (In the following, a bar on top of an $\mathcal {O}$-module will usually mean the module modulo its $\mathcal {O}$-torsion.) Write $H=H^*_!(X_U,M_{\mu ,\mathcal {O}}).$ There is an obvious surjection

10 $$\begin{aligned} \mathbb {T}^S(H) \rightarrow \mathbb {T}^S((\overline{H})_\mathfrak {m}). \end{aligned}$$

Let $t\in \mathbb {T}^S(H)$. Since *H* is a finitely generated $\mathcal {O}$-module and $(\overline{H})_\mathfrak {m}$ is a quotient of it, we know that *t* is in the kernel of (10) iff there exists $a\in \mathbb {N}, b\in \mathbb {T}^S-\mathfrak {m}$ such that $\varpi ^a bt=0$, which is equivalent to $t \in \ker (\mathbb {T}^S(H)\twoheadrightarrow \mathbb {T}^S(H)_\mathfrak {m}/\mathcal {O}\text {-tors})$. Thus (10) induces the desired isomorphism.

### Congruence ideals for automorphic representations

The previous lemma means we are now in the situation of section 3.

#### Definition 4.4

With the setup above, define the *congruence ideal*

$$\begin{aligned} \eta _{\pi }:=\eta _\lambda =\lambda (Ann_T(\ker \lambda ))=Fitt_\mathcal {O}\left( \frac{eT}{eT\cap T}\right) \end{aligned}$$

as in definitions 3.1, 3.2, where *e* is the idempotent in $T\otimes _\mathcal {O}E$ corresponding to (1, 0) in the decomposition $T\otimes _\mathcal {O}E \cong E \times T^c_E$ induced by $\lambda$. For $i\in \{b, t\}$ and a permissible $\epsilon \in \widehat{K_\infty /K_\infty ^\circ }$, define the *cohomological congruence ideal*

$$\begin{aligned} \eta _{\pi ,i,\epsilon }:=\eta _{\lambda }(H^i_!(X_U,M_{\mu ,\mathcal {O}})_\mathfrak {m}[\epsilon ]/\mathcal {O}\text {-torsion}) = Fitt_\mathcal {O}\left( \frac{eH}{eH\cap H}\right) \end{aligned}$$

where $$H=H^i_!(X_U,M_{\mu ,\mathcal {O}})_\mathfrak {m}[\epsilon ]/\mathcal {O}\text {-torsion}$$.

By the uniqueness of local new vectors [15, Theorem 11.5.6], we can pick a compact open $U=\prod _{v\not \mid \infty }U_v \le \prod _{v\not \mid \infty }\textrm{GL}_n(\mathcal {O}_{F_v})$ such that $(\pi ^{\infty })^U$ is one dimensional. Fix such *U*. We shall assume that *U* is a neat and hence a good subgroup (definition 2.1).

#### Lemma 4.5

Fix $i\in \{b, t\}$ and a permissible $\epsilon \in \widehat{K_\infty /K_\infty ^\circ }$. Let $p>2$ and $H=H^i_!(X_U,M_{\mu ,\mathcal {O}})_\mathfrak {m}[\epsilon ]/\mathcal {O}\text {-torsion}$.

- Then $H[\ker \lambda ]$ is a free $\mathcal {O}$-module of rank one whose base change to $\mathbb {C}$ is canonically isomorphic to
  $$\begin{aligned} H^i_!(X_U,M_{\mu ,\mathbb {C}})[(\pi ^{\infty ,S})^{U^S}\times \epsilon ] \end{aligned}$$
  as a $\mathbb {C}$-vector space.
- $\eta _{\pi ,i,\epsilon }\supset \eta _\pi .$ Equality holds if *H* is a free *T*-module of rank 1.

#### Proof

For part (a), we know $T[\ker \lambda ]$ is a finite free $\mathcal {O}$-module. To find its rank, note that $\ker \lambda$ is the image of $\ker \Lambda =(t-\Lambda (t):t\in \mathcal \mathbb {T}^S)$ under the projection $q:\mathbb {T}^S\twoheadrightarrow T$. As *T* is Noetherian, we can pick $t_1,\dots ,t_n\in \mathbb {T}^S$ such that $q((t_i-\Lambda (t_i):1\le i\le n))=\ker \lambda$. Then

$$\begin{aligned} H[\ker \lambda ]=H[\{t_i-\Lambda (t_i):1\le i\le n\}]=\ker (H\xrightarrow {m\mapsto (t_i-\Lambda (t_i)m)} H^n). \end{aligned}$$

Taking kernel commutes with flat base change, so

11 $$\begin{aligned} H[\ker \lambda ]\otimes _\mathcal {O}\mathbb {C}&= (H^i_!(X_U,M_{\mu ,\mathcal {O}})_\mathfrak {m}[\epsilon ]\otimes _\mathcal {O}\mathbb {C})[\ker \lambda ]\nonumber \\&= H^i_!(X_U,M_{\mu ,\mathbb {C}})_\mathfrak {m}[\epsilon ][\ker \lambda ] \nonumber \\&=H^i_!(X_U,M_{\mu ,\mathbb {C}})[\epsilon ][\ker \lambda ]\end{aligned}$$

12 $$\begin{aligned}&=H^i_!(X_U,M_{\mu ,\mathbb {C}})[(\pi ^{\infty ,S})^{U^S}][\epsilon ]\nonumber \\&=H^i(\mathfrak g,K_\infty ^\circ ,\pi _\infty \otimes _\mathbb {C}M_{\mu ,\mathbb {C}})[\epsilon ]\otimes _\mathbb {C}(\pi ^\infty )^U. \end{aligned}$$

Equation (11) holds because $H^i_!(X_U,M_{\mu ,\mathbb {C}})_\mathfrak {m}=H^i_!(X_U,M_{\mu ,\mathbb {C}})[\mathfrak {m}^\infty ]$ by [19, section 2.5.1]. Equation (12) holds by Lemma 2.16. By Lemma 2.12 and the choice of *U*, (12) is a one dimensional $\mathbb {C}$-vector space. This proves part (a).

The first part of (b) now follows from Lemma 3.4. The remaining part is easy. $\square$

### Betti-Whittaker periods

We shall mostly follow [1] to define the Betti-Whittaker period for this subsection. Fix all Haar measures as in that paper. Let $F,\pi ,\dots$ be defined as before.

#### Definition 4.6

Let $i\in \{b_n,t_n\}$. Fix a generator $w_\infty$ (depends on *i*) of the 1-dimensional space (Lemma 2.12)

$$\begin{aligned} H^{i}(\mathfrak g, K_\infty ^0,\pi _\infty \otimes M_{\mu ,\mathbb {C}})[\epsilon ]. \end{aligned}$$

Fix a continuous unitary homomorphism $\psi :F\backslash \mathbb {A}_F\rightarrow \mathbb {C}^\times$ such that $\psi _v$ is non-trivial^16^for all *v*. We shall abuse notation and let $\psi$ also denote the corresponding standard character on the unipotent radical of *G*, i.e. $\psi (u)=\psi (u_{1,2}+u_{2,3}+\dots + u_{n-1,n})$. Let $W(\pi ^\infty )$ be the Whittaker model of $\pi ^\infty$ with respect to $\psi ^\infty$. Let $V_\pi$ be the subspace^17^ of $L^2_0(G(F)\backslash G(\mathbb {A}_F),\chi )$ realizing $\pi$. Define $\mathcal {F}_{\pi ^\infty ,\epsilon ,w_\infty ,i}$ as the composition of the isomorphisms

$$\begin{aligned} W(\pi ^\infty )^U&\xrightarrow {\sim } W(\pi ^\infty )^U\otimes _\mathbb {C}H^{i}(\mathfrak g, K_\infty ^0,W(\pi _\infty )\otimes M_{\mu ,\mathbb {C}})[\epsilon ] \\&\xrightarrow {\sim } H^{i}(\mathfrak g, K_\infty ^0,W(\pi )^U\otimes M_{\mu ,\mathbb {C}})[\epsilon ] \\&\xrightarrow {\sim } H^{i}(\mathfrak g, K_\infty ^0,V_\pi ^U\otimes M_{\mu ,\mathbb {C}})[\epsilon ]\\&= H^{i}_{cusp}(X_U,M_{\mu ,\mathbb {C}})[(\pi ^\infty )^U\times \epsilon ]\\&\xrightarrow {\sim } H^i_!(X_U,M_{\mu ,\mathbb {C}})[(\pi ^{\infty ,S})^{U^S}\times \epsilon ]. \end{aligned}$$

The first map is $w^\infty \mapsto w^\infty \otimes w_\infty$; the second map is trivial; the third map is the inverse of the map

$$\begin{aligned} V_\pi&\xrightarrow {\sim } W(\pi )\\ f&\mapsto \left( g\mapsto \int _{U_n(F)\backslash U_n(\mathbb {A}_F)} f(ug)\psi ^{-1}(u)du\right) \end{aligned}$$

and $U_n$ is the unipotent radical of the standard Borel subgroup $B_n$. The last isomorphism is by Lemma 2.16.

#### Definition 4.7

For each finite place *v*, let $w_v$ be the essential vector^18^ of $\pi _v$ in its $\psi _v$-Whittaker model. Let $w^\infty =\otimes _{v<\infty } w_v$. Then $w^\infty \in W(\pi ^\infty )^U$. Let $H=H^i_!(X_U,M_{\mu ,\mathcal {O}})_\mathfrak {m}[\epsilon ]/\mathcal {O}\text {-torsion}$. By Lemma 4.5, $H[\ker \lambda ]\otimes _\mathcal {O}\mathbb {C}= H^i_!(X_U,M_{\mu ,\mathbb {C}})[(\pi ^{\infty ,S})^{U^S}\times \epsilon ].$ We define the *period*

$$\begin{aligned} \mathfrak p_{\pi ,i,\epsilon } \end{aligned}$$

to be the number in $\mathbb {C}^\times$ such that $\mathcal {F}_{\pi ^\infty ,\epsilon ,w_\infty ,i}(w^\infty )/\mathfrak p_{\pi ,i,\epsilon }$ is an $\mathcal {O}$-generator of $H[\ker \lambda ].$ This is well defined up to multiplication by $\mathcal {O}^\times$ by Lemma 4.5(a).

### $L(1,\pi ,{{\,\textrm{Ad}\,}}^\circ )$ and congruence ideals

Recall that $U_v=\textrm{GL}_n(\mathcal {O}_v)$ for all finite $v\notin S$. Let

$$\begin{aligned} \theta : \mathcal {H}^S(G^S,U^S)&\rightarrow \mathcal {H}^S(G^S,U^S)\\ [U^S g U^S]&\mapsto [U^S g^{-1} U^S]. \end{aligned}$$

This is an $\mathcal {O}$-algebra isomorphism.^19^

#### Lemma 4.8

The Hecke eigensystem $\tilde{\Lambda }:\mathbb {T}^S \rightarrow \mathcal {O}$ attached to the contragredient $\tilde{\pi }$ is given by $\Lambda \circ \theta$.

#### Proof

It is enough to show the analogous fact after base changing from $\mathcal {O}$ to $\mathbb {C}$ and working at a single place. Let $v\notin S$ be a finite place, $G=\textrm{GL}_n(F_v)$, $K=\textrm{GL}_n(\mathcal {O}_{F_v})$, $V=\pi _v$.

It is well known that the restriction map induces an isomorphism

13 $$\begin{aligned} (\widetilde{V})^K \xrightarrow {\sim } \widetilde{V^K} \end{aligned}$$

so in particular $(\widetilde{V})^K$ is 1-dimensional.

Let $g\in G$. By the same proof as [20, Lemma 5.5.1 (c)], there exist $g_1,\dots ,g_m\in G$ such that $K g K= \sqcup _i g_i K = \sqcup K g_i$. For all $f\in (\widetilde{V})^K$ and $v\in V^K$,

$$\begin{aligned} ([KgK]f)(v)=(\sum g_i\cdot f)(v)=f(\sum g_i^{-1}v) = f([Kg^{-1}K]v)=\lambda ([Kg^{-1}K])f(v). \end{aligned}$$

By (13), $[KgK]f=\lambda ([Kg^{-1}K])f$, as desired. $\square$

#### Proposition 4.9

(Poincaré duality) Let $d=dim X_U$. The cup product induces a perfect pairing

$$\begin{aligned} {[}\,,\,]:H^i_c(X_U,M_\mu )/(\mathcal {O}-\text {tors}) \times H^{d-i}(X_U,M_\mu ^\vee )/(\mathcal {O}-\text {tors}) \rightarrow \mathcal {O}\end{aligned}$$

where^20^$M_\mu ^\vee ={{\,\textrm{Hom}\,}}_\mathcal {O}(M_\mu ,\mathcal {O}).$ If *S* is a finite set of finite places such that $U_v=\textrm{GL}_n(\mathcal {O}_v)$ for all finite $v\notin S$, then

$$\begin{aligned} {[}tx,y] = [x, \theta (t)y] \end{aligned}$$

for all $$t\in \mathcal {H}^S(G^S,U^S)$$, $$x\in H^i_c(X_U,M_\mu )/(\mathcal {O}-\text {tors})$$, $$y\in H^{d-i}(X_U,M_\mu ^\vee )/(\mathcal {O}-\text {tors})$$.

A version of this is proved in [21, Theorem 4.8.9], but the proof is not that easy. We shall deduce this from Verdier duality instead.

#### Proof

As stated in [7, Proposition 2.2.20], we have by Verdier duality an isomorphism

$$\begin{aligned} RHom_\mathcal {O}(R\Gamma _c(X_U,M_\mu ),\mathcal {O}) \cong R\Gamma (X_U,M_\mu ^\vee )[d] \end{aligned}$$

in the derived category of $\mathcal {O}$-modules $D(\mathcal {O})$. It follows from one of the spectral sequences for *Ext* that we have a spectral sequence

$$\begin{aligned} E_2^{i,j}=Ext_\mathcal {O}^i(H^{-j}_c(X_U,M_\mu ),\mathcal {O})\Rightarrow E^{i+j}=H^{i+j+d}(X_U,M_\mu ^\vee ). \end{aligned}$$

Since $\mathcal {O}$ is a PID, the only non-zeros terms lie in $\{(i,j):0\le i \le 1, -d\le j\le 0\}$. For all $j\in \mathbb {Z}$, we have an exact sequence

$$\begin{aligned} 0\rightarrow E_2^{1,-j-1} \rightarrow E^{-j} \rightarrow E_2^{0,-j} \rightarrow 0 \end{aligned}$$

i.e.

$$0 \rightarrow Ext_\mathcal {O}^1(H^{j+1}_c(X_U,M_\mu ),\mathcal {O}) \rightarrow H^{d-j}(X_U,M_\mu ^\vee ) \xrightarrow {f} {{\,\textrm{Hom}\,}}_\mathcal {O}(H^{j}_c(X_U,M_\mu ),\mathcal {O}) \rightarrow 0.$$

Since $H^{j+1}_c(X_U,M_\mu )$ is finitely generated over $\mathcal {O}$ by the existence of Borel-Serre compactification, the second term is $\mathcal {O}$-torsion. On the other hand, the 4th term is $\mathcal {O}$-torsion free. It follows that $\ker f$ is precisely the $\mathcal {O}$-torsion of $H^{d-j}(X_U,M_\mu ^\vee )$, so *f* induces an isomorphism

$$\tilde{f}:H^{d-j}(X_U,M_\mu ^\vee )/(\mathcal {O}-\text {tors}) \xrightarrow {\sim } {{\,\textrm{Hom}\,}}_\mathcal {O}(H^{j}_c(X_U,M_\mu )/(\mathcal {O}-\text {tors}),\mathcal {O}).$$

We have a pairing

$$\begin{aligned} H^{d-j}(X_U,M_\mu ^\vee )/(\mathcal {O}-\text {tors})&\times H^{j}_c(X_U,M_\mu )/(\mathcal {O}-\text {tors}) \rightarrow \mathcal {O}\\ (a&,b)\mapsto \tilde{f}(a)(b). \end{aligned}$$

This is a perfect pairing because both $H^{d-j}(X_U,M_\mu ^\vee )/(\mathcal {O}-\text {tors})$ and $H^{j}_c(X_U,M_\mu )/(\mathcal {O}-\text {tors})$ are finite free $\mathcal {O}$-modules and $\tilde{f}$ is an isomorphism. It is well-known that this is given by the cup product. The last assertion about the action of the Hecke algebra follows from [7, Proposition 2.2.20]. $\square$

The cup product induces a pairing

$$\begin{aligned} {[}\,,\,]:H^i_!(X_U,M_\mu )/(\mathcal {O}-\text {tors}) \times H^{d-i}_!(X_U,M_\mu ^\vee )/(\mathcal {O}-\text {tors}) \rightarrow \mathcal {O}. \end{aligned}$$

For convenience, let

$$\begin{aligned} \overline{H}^i_!(X_U,M_\mu ):= H^i_!(X_U,M_\mu )/(\mathcal {O}-\text {tors}) \end{aligned}$$

and $\overline{H}^{d-i}_!(X_U,M_\mu ^\vee ):=H^{d-i}(X_U,M_\mu ^\vee )/(\mathcal {O}-\text {tors})$. Let $\partial X_U$ denote the boundary of the Borel-Serre compactification of $X_U$. Let $\tilde{\mathfrak {m}}=\theta (\mathfrak {m})\subset \mathbb {T}^S$, which equals $\ker (\widetilde{\Lambda }\mod \varpi )$ by Lemma 4.8, where $\tilde{\Lambda }:\mathbb {T}^S \rightarrow \mathcal {O}$ is the Hecke eigensystem attached to the contragredient $\tilde{\pi }$. Let $\tilde{\epsilon }: K_\infty /K_\infty ^\circ \rightarrow \{\pm 1\}$ be the character such that for every real place *v*, if $x_v\in K_v/K_v^\circ$ is non-trivial, then $\tilde{\epsilon }(x_v)=(-1)^{n-1}\epsilon (x_v)$.

#### Corollary 4.10

Assume $H^b(\partial X_U,M_\mu )_\mathfrak {m}$ is $\mathcal {O}$-torsion free and $p>2$.

- Then
  $$\begin{aligned} {[}\,,\,]:\overline{H}^b_!(X_U,M_\mu )_\mathfrak {m}\times \overline{H}^{t}_!(X_U,M_\mu ^\vee )_{\tilde{\mathfrak {m}}} \rightarrow \mathcal {O}\end{aligned}$$
  and
  $$\begin{aligned} {[}\,,\,]:\overline{H}^b_!(X_U,M_\mu )_\mathfrak {m}[\epsilon ] \times \overline{H}^{t}_!(X_U,M_\mu ^\vee )_{\tilde{\mathfrak {m}}}[\tilde{\epsilon }] \rightarrow \mathcal {O}\end{aligned}$$
  are both perfect pairings.
- $\theta$ induces^21^ an isomorphism $\mathbb {T}^S(\overline{H}^b_!(X_U,M_\mu )_\mathfrak {m})\cong \mathbb {T}^S(\overline{H}^t_!(X_U,M_\mu ^\vee )_{\tilde{\mathfrak {m}}}).$

#### Proof

Let $q_1:\mathbb {T}^S \rightarrow \mathbb {T}^S(\overline{H}^b_!(X_U,M_\mu ))$ and $q_2:\mathbb {T}^S \rightarrow \mathbb {T}^S(\overline{H}^{t}_!(X_U,M_\mu ^\vee ))$ be the quotient maps. Note that the image of these two maps are finite $\mathcal {O}$-algebras, so they are product of local rings.

#### Claim

For $i=1,2$, let $m_i \subset \mathbb {T}^S$ be maximal ideals containing $\ker q_i$. Then $[\overline{H}^b_!(X_U,M_\mu )_{\mathfrak {m}_1}, \overline{H}^{t}_!(X_U,M_\mu ^\vee )_{\mathfrak {m}_2}]=0$ unless $\mathfrak {m}_2=\theta (\mathfrak {m}_1)$.

To show this, we let $A:= H^b_!(X_U,M_{\mu ,\mathbb {C}})_{\mathfrak {m}_1}, B:= H^{t}_!(X_U,M_{\mu ,\mathbb {C}}^\vee )_{\mathfrak {m}_2}$. Suppose $[A,B]\ne 0$. By [19, section 2.5.1],

14 $$\begin{aligned} A=H^b_!(X_U,M_{\mu ,\mathbb {C}})[\mathfrak {m}_1^\infty ]. \end{aligned}$$

By Lemma 2.16, $\mathbb {T}^S_\mathbb {C}$ acts semisimply on $H^b_!(X_U,M_{\mu ,\mathbb {C}}).$ As $\mathbb {T}^S$ is commutative, every simple $\mathbb {T}^S_\mathbb {C}$-submodule of $H^b_!(X_U,M_{\mu ,\mathbb {C}})$ is 1 dimensional. It follows that with respect to a suitable basis of $H^b_!(X_U,M_{\mu ,\mathbb {C}})$, every element of $\mathbb {T}^S_\mathbb {C}$ acts on $H^b_!(X_U,M_{\mu ,\mathbb {C}})$ by a diagonal matrix. Hence by (14), $A=H^b_!(X_U,M_{\mu ,\mathbb {C}})[\mathfrak {m}_1]$. Similarly, $B = H^{t}_!(X_U,M_{\mu ,\mathbb {C}}^\vee )[\mathfrak {m}_2].$

By assumption, there exists $a\in A$ such that $[a,B]\ne 0$. Let $t\in \mathfrak {m}_1$. For all $b\in B$,

$$\begin{aligned} 0=[ta,b]=[a,\theta (t)b], \end{aligned}$$

so $\theta (t)$ is not surjective as an endomorphism on *B* and hence not injective, so there exists $c\in B-\{0\}$ such that $\theta (t)c=0$. As $\mathfrak {m}_2$ is a maximal ideal of $\mathbb {T}^S$, the annihilators of *c* in $\mathbb {T}^S$ is $\mathfrak {m}_2$, so $\theta (t)\in \mathfrak {m}_2$. This means $\theta (\mathfrak {m}_1)\subset \mathfrak {m}_2$, so $\theta (\mathfrak {m}_1)=\mathfrak {m}_2$. This proves the claim.

We know that

$$\begin{aligned} \overline{H}^b_!(X_U,M_\mu ) = \bigoplus _{\begin{array}{c} \mathfrak {m}_1\lhd \mathbb {T}^S \\ \mathfrak {m}_1\supset \ker q_1 \end{array}}\overline{H}^b_!(X_U,M_\mu )_{\mathfrak {m}_1} \end{aligned}$$

and similarly for $\overline{H}^{t}_!(X_U,M_\mu ^\vee )$. Using this and the claim, we can deduce the first part of (a) by the same argument as [1, section 4.2.4] under our assumption that $H^b(\partial X_U,M_\mu )_\mathfrak {m}$ is $\mathcal {O}$-torsion free. Let us just remark that, $H^b(\partial X_U,M_\mu )_\mathfrak {m}$ appears because we have a long exact sequence $\dots \rightarrow H^i_c(X_U,M_\mu )\rightarrow H^i(X_U,M_\mu ) \rightarrow H^i(\partial X_U,M_\mu )\rightarrow \dots$

For the second part of (a), note that we have a decomposition

$$\begin{aligned} \overline{H}^b_!(X_U,M_\mu )_\mathfrak {m}= \bigoplus _{\epsilon _1\in \widehat{K_\infty /K_\infty ^\circ }}\overline{H}^b_!(X_U,M_\mu )_{\mathfrak {m}}[\epsilon _1] \end{aligned}$$

since $p>2$. The perfectness then follows from the first part and the proof of [1, Proposition 3.3.1]. (The proof there works here in view of the decomposition of $H^{i}(\mathfrak g, K_\infty ^0,\pi _\infty \otimes _\mathbb {C}M_{\mu ,\mathbb {C}})$ in the last part of the proof of 2.12.)

Part (b) follows from part (a) and the same argument as [7, Corollary 2.2.21], namely the commutativity of the diagram 
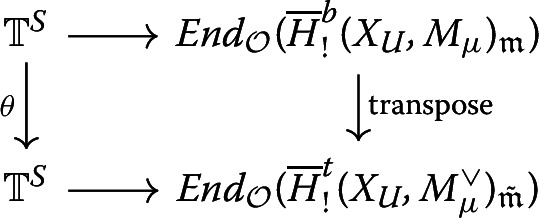
$$\square $$

#### Lemma 4.11

Let $\phi ,\tilde{\phi }$ be cusp forms in the space of cusp forms affording $\pi ,\tilde{\pi }$ respectively. Define

$$\begin{aligned} \langle \phi ,\tilde{\phi } \rangle = \int _{A_G G(F)\backslash G(\mathbb {A}_F)} \phi (g)\tilde{\phi }(g) dg. \end{aligned}$$

Then

$$\begin{aligned} \langle \phi ,\tilde{\phi } \rangle = \frac{\prod _{v\mid \infty }\mathfrak c_v^\sharp (w_v,\tilde{w}_v) \cdot L(1,\pi ,{{\,\textrm{Ad}\,}}^\circ )}{\alpha _F\; \mathfrak p_{ram}(\pi )}, \end{aligned}$$

where $\alpha _F:=\frac{\hat{\Phi }_f(0)}{n {{\,\textrm{Res}\,}}_{s=1}\tilde{\zeta }_F(s)}$ with $\Phi _f$ the characteristic function of $\prod _{v\not \mid \infty }\mathcal {O}_{F_v}^n$, $\hat{\Phi }_f$ its Fourier transform, and $\tilde{\zeta }_F$ the completed zeta function. The measures, $\mathfrak c_v^\sharp (w_v,\tilde{w}_v)$, and $\mathfrak p_{ram}(\pi )$ are defined as in [1, section 2]. Also, $L(1,\pi ,{{\,\textrm{Ad}\,}}^\circ )$ is the value at 1 of the Langlands *L*-function (see e.g. [15, section 12.7]).

#### Proof

Note that $\int _{A_G G(F)\backslash G(\mathbb {A}_F)} = \int _{Z(\mathbb {A}_F) G(F)\backslash G(\mathbb {A}_F)} \int _{A_G G(F)\backslash Z(\mathbb {A}_F)G(F)}$, where *Z* is the centre of *G*. Also, $A_G G(F)\backslash Z(\mathbb {A}_F)G(F) = F^\times \backslash \mathbb {A}^1_F$, where $\mathbb {A}^1_F:=\{x\in \mathbb {A}_F: |x|_{\mathbb {A}_F}=1\}.$ It follows that

$$\begin{aligned} \langle \phi ,\tilde{\phi } \rangle = vol(F^\times \backslash \mathbb {A}_F^1)\int _{Z(\mathbb {A}_F)G(F)\backslash G(\mathbb {A}_F)} \phi (g)\tilde{\phi }(g) dg. \end{aligned}$$

The result now follows from [1, equation (2.2.11)]. They obtained their result by relating the Petersson inner product with $L(1,\pi ,{{\,\textrm{Ad}\,}}^0)$ by the Rankin-Selberg method and using the fact that $L(s,\pi \times \tilde{\pi })=\tilde{\zeta }_F(s)L(s,\pi ,{{\,\textrm{Ad}\,}}^0).$ $\square$

#### Lemma 4.12

We have

$$\begin{aligned} {[}\vartheta ^\circ _{b,\epsilon },\tilde{\vartheta }^\circ _{t,\tilde{\epsilon }}] = L^{alg}(1,\pi ,{{\,\textrm{Ad}\,}}^0,\epsilon ), \end{aligned}$$

where $[,\;]$ is the pairing induced by cup product as before, $\vartheta ^\circ _{b,\epsilon }$ is an $\mathcal {O}$-basis of $\overline{H}^b_!(X_U,M_\mu )_\mathfrak {m}[\epsilon ][\ker \lambda ]$, $\tilde{\vartheta }^\circ _{t,\tilde{\epsilon }}$ is an $\mathcal {O}$-basis of $\overline{H}^t_!(X_U,M_\mu ^\vee )_{\tilde{\mathfrak {m}}}[\tilde{\epsilon }][\ker \tilde{\lambda }]$, and

$$\begin{aligned} L^{alg}(1,\pi ,{{\,\textrm{Ad}\,}}^0,\epsilon ):=\frac{L(1,\pi ,{{\,\textrm{Ad}\,}}^0)}{\alpha _F \,\mathfrak p_{ram}(\pi )\mathfrak p_\infty (\pi )\mathfrak p_{\pi ,b,\epsilon }\mathfrak p_{\tilde{\pi },t,\tilde{\epsilon }}}. \end{aligned}$$

Here, $\mathfrak p_\infty (\pi )$ is defined as in [1, equation (3.3.9)] and $\mathfrak p_{\pi ,b,\epsilon }, \mathfrak p_{\tilde{\pi },t,\tilde{\epsilon }}$ are defined in Definition 4.7.^22^

#### Proof

This is more or less what [1, section 3.3.3] obtained, except that our space $X_U$ is different from the locally symmetric spaces they used. As in [1, p.658], we have

$$\begin{aligned} {[}\mathfrak p_{\pi ,b,\epsilon }\vartheta ^\circ _{b,\epsilon },\mathfrak p_{\tilde{\pi },t,\tilde{\epsilon }}\tilde{\vartheta }^\circ _{t,\tilde{\epsilon }}]&= \frac{1}{vol(U)} \int _{G(F) \backslash G(\mathbb {A}_F)/K_\infty ^\circ U} \varsigma \\&= \frac{1}{vol(U)}\int _{G(F) \backslash G(\mathbb {A}_F)/A_G U} \varsigma \\&= \int _{G(F) \backslash G(\mathbb {A}_F)/A_G} \varsigma \\&= \frac{L(1,\pi ,{{\,\textrm{Ad}\,}}^0)}{\alpha _F \,\mathfrak p_{ram}(\pi )\mathfrak p_\infty (\pi )} \end{aligned}$$

where $\varsigma$ has the same meaning as that in [1, p.658] and in the last equality we used Lemma 4.11 instead of [1, equation (2.2.11)]. Dividing both sides by $\mathfrak p_{\pi ,b,\epsilon }\mathfrak p_{\tilde{\pi },t,\tilde{\epsilon }}$ gives the result. $\square$

#### Remark 4.13

$\alpha _F$ depends only on *F*, $\mathfrak p_{ram}(\pi )$ depends only on the ramified components of $\pi$, and $\mathfrak p_{\infty }(\pi )$ depends only on $\pi _\infty$.

We can now prove our first main theorem.

#### Theorem 4.14

Let the notation be as at the start of Section 4. Suppose that $H^b(\partial X_U,M_\mu )_\mathfrak {m}$ is $\mathcal {O}$-torsion free. Then

$$\begin{aligned} \eta _{\pi ,b,\epsilon }=\eta _{\tilde{\pi },t,\tilde{\epsilon }}=(L^{alg}(1,\pi ,{{\,\textrm{Ad}\,}}^0,\epsilon )). \end{aligned}$$

#### Proof

Since $H^b(\partial X_U,M_\mu )_\mathfrak {m}$ is $\mathcal {O}$-torsion free, the cup product gives a perfect pairing

$$\begin{aligned} {[}\,,\,]:\overline{H}^b_!(X_U,M_\mu )_\mathfrak {m}[\epsilon ] \times \overline{H}^{t}_!(X_U,M_\mu ^\vee )_{\tilde{\mathfrak {m}}}[\tilde{\epsilon }] \rightarrow \mathcal {O}\end{aligned}$$

by Corollary 4.10.^23^ We would like to apply Lemma 3.5. The conditions in part (b) of that lemma is satisfied by Lemma 4.5 and the fact that $(\pi ^\infty )^U$ and $(\tilde{\pi }^\infty )^U$ are one dimensional.

Recall that $\lambda :T\rightarrow \mathcal {O}$ is the Hecke eigensystem for $\pi$. This factors through $\mathbb {T}^S(\overline{H}^b_!(X_U,M_\mu )_\mathfrak {m})$ because the action of the Hecke algebra on $\overline{H}^*_!(X_U,M_\mu )_\mathfrak {m}$ preserves degree and $\pi$ is isomorphic to a submodule of $\overline{H}^b_!(X_U,M_\mu )_\mathfrak {m}\otimes _\mathcal {O}\mathbb {C}$.

Similar to how we defined the idempotent $e\in T_K$ using $\lambda :T\rightarrow \mathcal {O}$, we can define an idempotent $e'\in \mathbb {T}^S(\overline{H}^b_!(X_U,M_\mu )_\mathfrak {m})_K$ using the induced map on the quotient $\mathbb {T}^S(\overline{H}^b_!(X_U,M_\mu )_\mathfrak {m})\rightarrow \mathcal {O}$. It is clear from the definitions of *e* and $e'$ that $e'$ is the image of *e* under $\mathbb {T}\twoheadrightarrow \mathbb {T}^S(\overline{H}^b_!(X_U,M_\mu )_\mathfrak {m})$.

The same argument (with $H^b_!(X_U,M_\mu )_\mathfrak {m}$ replaced by $H^t_!(X_U,M_\mu ^\vee )_{\tilde{\mathfrak {m}}}$) works for the contragredient $\tilde{\pi }$, $\tilde{\lambda }$, $\tilde{e}, \tilde{e}'$ and we know $\tilde{e}'$ is the image of $\tilde{e}$ under $\tilde{T} \twoheadrightarrow \mathbb {T}^S(\overline{H}^t_!(X_U,M_\mu ^\vee )_{\tilde{\mathfrak {m}}})$.

By Lemma 4.8, we know $\tilde{\lambda }=\lambda \circ \theta$. It follows from definition and part (b) of Corollary 4.10 that $\theta (e')=\theta (\tilde{e}')$. Thus, for all $x\in \overline{H}^b_!(X_U,M_\mu )_\mathfrak {m}[\epsilon ]$, $y \in \overline{H}^{t}_!(X_U,M_\mu ^\vee )_{\tilde{\mathfrak {m}}}[\tilde{\epsilon }]$, we have

$$\begin{aligned} {[}ex,y]=[e'x,y]=[x,\theta (e')y]=[x,\tilde{e}' y]=[x,\tilde{e} y]. \end{aligned}$$

From this^24^ and the fact that $e,e'$ are idempotents, we deduce that all the conditions of Lemma 3.5 are satisfied.

By Lemma 3.5 and Lemma 4.12,

$$\begin{aligned} \eta _{\pi ,b,\epsilon }=\eta _{\tilde{\pi },t,\tilde{\epsilon }}= [\vartheta ^\circ _{b,\epsilon },\tilde{\vartheta }^\circ _{t,\tilde{\epsilon }}] =(L^{alg}(1,\pi ,{{\,\textrm{Ad}\,}}^0,\epsilon )). \end{aligned}$$

$$\square $$

#### Remark 4.15

The theorem is a generalisation of [4, Proposition 4.12 first part and Lemma 5.6(iv)], where analogous results were obtained for $\textrm{GL}_2$ over a totally real field and over an imaginary quadratic field.^25^

An analogous result is stated in [1, section 4]. It differs from our result in the following ways: the result there is for the product of $L^{alg}(1,\pi ,{{\,\textrm{Ad}\,}}^0,\epsilon )$ over all permissible $\epsilon$ while our result is for each individual permissible $\epsilon$. Additionally, we localize at a maximal ideal throughout, which is necessary for relating congruence ideals to Selmer groups (see Theorem 4.26 below). Localisation also makes some results slightly harder to prove, but requires a weaker hypothesis that $H^b(\partial X_U,M_\mu )_\mathfrak {m}$, rather than $H^b(\partial X_U,M_\mu )$, is $\mathcal {O}$-torsion free (see Lemma 4.19 below for a case where this holds). Although this weaker hypothesis was mentioned in [1, page 669], the necessary (small) adjustments to their proof were not worked out. We have provided more detailed arguments for certain parts. Furthermore, to facilitate the use of some results in [7] and [14], we work with a different locally symmetric space, preventing us from applying some results from [1] directly. For the same reason, our $L^{alg}(1,\pi ,{{\,\textrm{Ad}\,}}^0,\epsilon )$ is slightly different from theirs.

#### Remark 4.16

It may be possible to determine $\mathfrak p_\infty (\pi )$ explicitly as a power of $2\pi i$ using techniques of [22], but we have not attempted this. In that paper, they precisely determined some archimedean zeta-integrals by replacing $\pi$ with simpler automorphic representations $\pi '$ with $\pi _\infty \cong \pi _\infty '$. Here, ’simpler’ means $\pi '$ is automorphically induced from a Hecke character or is an isobaric sum of Hecke characters. This approach allows them to relate the *L*-function of $\pi$ to those of Hecke characters, which, in turn, are related to CM periods by results of Blasius.

Now, we will explain why this is related to congruences of automorphic representations. Roughly speaking, if $L^{alg}(1,\pi ,{{\,\textrm{Ad}\,}}^0,\epsilon )$ is not a *p*-adic unit, then $\pi$ is congruent to another automorphic representation. The converse holds if the maximal ideal $\mathfrak {m}$ is non-Eisenstein.

#### Corollary 4.17

Let us keep the same assumptions as Theorem 4.14. Consider the following two statements:

- $$\varpi \mid L^{alg}(1,\pi ,{{\,\textrm{Ad}\,}}^0,\epsilon )$$.
- There is a discrete automorphic representation $\pi '\not \cong \pi$ of $\textrm{GL}_n(\mathbb {A}_F)$ with $H^b_!(X_U,M_{\mu ,\mathbb {C}})[(\pi '^{\infty ,S})^{U^S}]\ne 0$ whose Hecke eigensystem^26^$\Lambda ':\mathbb {T}^S\rightarrow \overline{\mathbb {Q}}_p$ satisfies $|\Lambda (t)-\Lambda '(t)|_p<1$ for all $t\in \mathbb {T}^S$.

Then (a) implies (b). If $H^b_!(X_U,M_{\mu ,\mathcal {O}})_\mathfrak {m}[\epsilon ]/\mathcal {O}\text {-torsion}$ is a free *T*-module, then (b) implies (a).

Note that $\pi '$ needs not be cuspidal even though we start with a cuspidal $\pi$. See Corollary 4.20 below however.

#### Proof

Abusing notation, we shall identify $\overline{\mathbb {Q}}_p$ with $\mathbb {C}$ using our fixed isomorphism $\iota$. Note that $\pi '\cong \pi$ iff $\Lambda '=\Lambda$ by the strong multiplicity one theorem (where we regard $\Lambda$ as having codomain in $\overline{\mathbb {Q}}_p$).

Suppose $\varpi \mid L^{alg}(1,\pi ,{{\,\textrm{Ad}\,}}^0,\epsilon )$. By Theorem 4.14 and Lemma 3.4, $\eta _\pi \ne \mathcal {O}$. By Remark 3.7 there exists a $\overline{\mathbb {Q}}_p$-algebra homomorphism $\lambda ':T\otimes _\mathcal {O}\overline{\mathbb {Q}}_p \rightarrow \overline{\mathbb {Q}}_p$ such that $\lambda '\ne \lambda \otimes _{\mathcal {O}}\overline{\mathbb {Q}}_p$ and $|\lambda (t)-\lambda '(t)|<1$ for all $t\in T$. We have a natural map

$$\begin{aligned} \mathbb {T}^S(H^*_!(X_U,M_\mu ))\otimes _\mathcal {O}\overline{\mathbb {Q}}_p = \mathbb {T}^S_{\overline{\mathbb {Q}}_p}(V)\rightarrow T\otimes _\mathcal {O}\overline{\mathbb {Q}}_p, \end{aligned}$$

where $V:=H^*_!(X_U,M_{\mu ,\overline{\mathbb {Q}}_p})$ and $\mathbb {T}^S_{\overline{\mathbb {Q}}_p}(V):= im(\mathbb {T}^S\otimes _\mathcal {O}\bar{\mathbb {Q}}_p \rightarrow {{\,\textrm{End}\,}}_{\bar{\mathbb {Q}}_p}(V))$. Composing this with $\lambda '$, we get a $\overline{\mathbb {Q}}_p$-algebra homomorphism

$$\begin{aligned} f:\mathbb {T}^S_{\overline{\mathbb {Q}}_p}(V)\rightarrow \overline{\mathbb {Q}}_p. \end{aligned}$$

Let $$\mathfrak {n}:=\ker f$$. Then

$$\begin{aligned} V_\mathfrak {n}\ne 0 \end{aligned}$$

because $Supp_{\mathbb {T}^S_{\overline{\mathbb {Q}}_p}(V)}(V)= \{\mathfrak p\in Spec(\mathbb {T}^S_{\overline{\mathbb {Q}}_p}(V)): \mathfrak p\supset Ann_{\mathbb {T}^S_{\overline{\mathbb {Q}}_p}(V)}(V)\}=Spec(\mathbb {T}^S_{\overline{\mathbb {Q}}_p}(V)).$ By [19, section 2.5.1], $V[\mathfrak {n}]\ne 0$. By Lemma 2.16 part (a), there is a discrete automorphic representation $\pi '$ such that $(\pi '^{\infty ,S})^{U^S}$ is isomorphic to a sub-$\mathbb {T}^S_{\overline{\mathbb {Q}}_p}(V)$-module of *V* and $(\pi '^{\infty ,S})^{U^S}[\mathfrak {n}]\ne 0.$ Note that $(\pi '^{\infty ,S})^{U^S} = \otimes '_v (\pi '_v)^{U_v}$ is one dimensional over $\overline{\mathbb {Q}}_p$, so $(\pi '^{\infty ,S})^{U^S}[\mathfrak {n}]= (\pi '^{\infty ,S})^{U^S}.$ The Hecke eigensystem attached to $\pi '$ has the desired property.

Suppose $H^b_!(X_U,M_{\mu ,\mathcal {O}})_\mathfrak {m}[\epsilon ]/\mathcal {O}\text {-torsion}$ is a free *T*-module, say, of rank *d*, and there is a $\pi '$ satisfying the statement of the corollary. By freeness, $\eta _{\pi ,b,\epsilon }=\eta _\pi ^d$, so by Theorem 4.14, it suffices to show that $\eta _\pi \ne \mathcal {O}$. By Lemma 3.6, it suffices to show that there is an $\mathcal {O}$-algebra homomorphism $\lambda ':T\rightarrow \overline{\mathbb {Q}}_p$ with $\lambda \ne \lambda '$ and $|\lambda (t)-\lambda '(t)|<1$ for all $t\in T$. Equivalently, we need to show that there is an $\mathcal {O}$-algebra homomorphism $\Lambda '':\mathbb {T}^S\rightarrow \overline{\mathbb {Q}}_p$ that factors through $\mathbb {T}^S(H^*_!(X_U,M_{\mu }))$ with $\Lambda \ne \Lambda ''$ and $|\Lambda (t)-\Lambda ''(t)|<1$ for all $t\in \mathbb {T}^S$, because any such $\Lambda ''$ necessarily factors through *T*. For this, we can take $\Lambda ''$ to be the Hecke eigensystem attached to $\pi '$. $\square$

#### Remark 4.18

A version of Corollary 4.17 appears in [1, Theorem 4.3.1], but the conditional converse is not stated explicitly and is not proved. An analogue of Corollary 4.17 in the case of $\textrm{GL}_2$ is also proved in [23, Theorem 5.25] under certain conditions for minimal and ordinary eigenforms.

We think the condition for the converse, namely that $H^b_!(X_U,M_{\mu ,\mathcal {O}})_\mathfrak {m}[\epsilon ]/\mathcal {O}\text {-torsion}$ is a free *T*-module, is not unreasonable. When *F* is a CM field, it should be possible to verify this using the Taylor-Wiles method if $\mathfrak {m}$ is non-Eisenstein and the Galois representation attached to $\pi$ is a minimally ramified deformation of its residual representation. However, under our current knowledge of Galois representations, such an approach will require a lot of extra assumptions and conjectures (such as the vanishing of $H^i(X_U,k)_\mathfrak {m}$ outside the cuspidal range, existence of Hecke algebra valued Galois representations (without nilpotent ideals), local-global compatibility of such representations). Therefore, we do not pursue this approach here. See, however, [24] for the $\textrm{GL}_2$ case.^27^ It may also be possible to extend the freeness to the non-minimal case using the methods of [25, 26].

Now, assume in addition that *F* is a CM field that contains an imaginary quadratic field and *S* comes via pullback from a set of finite places of $\mathbb {Q}$ which contains *p* and all places at which $F/\mathbb {Q}$ is ramified. These conditions guarantee the existence of various Galois representations [27, section V.4]. We say that a maximal ideal $\mathfrak {m}$ of $\mathbb {T}^S(H^*(X_U,M_{\mu }))$ is *non-Eisenstein* if the residual Galois representation $\overline{\rho }_\mathfrak {m}:{{\,\textrm{Gal}\,}}(\overline{F}/F)\rightarrow \textrm{GL}_n(\overline{ \mathbb {F}}_p)$ attached to $\mathfrak {m}$ is absolutely irreducible.

#### Lemma 4.19

Let the notation be as above. Let $\mathfrak {m}$ be a non-Eisenstein maximal ideal of $\mathbb {T}^S(H^*(X_U,M_{\mu }))$. Then

- $$H^*(\partial X_U,M_{\mu })_\mathfrak {m}=0$$,
- $$H^*(X_U,M_{\mu })_\mathfrak {m}=H^*_!(X_U,M_{\mu })_\mathfrak {m}$$,
- $$T=\mathbb {T}^S(H^*(X_U,M_{\mu }))_{\mathfrak {m}}/\mathcal {O}\text {-tors}$$,
- $$H^*(X_U,M_{\mu ,\mathbb {C}})_\mathfrak {m}=H^*_!(X_U,M_{\mu ,\mathbb {C}})_\mathfrak {m}=H^*_{cusp}(X_U,M_{\mu ,\mathbb {C}})_\mathfrak {m}$$.

#### Proof

The key input for the first part is [14, Theorem 4.2], which states that for every smoooth $\mathcal {O}[U_S]$-module *A* that is finite as an $\mathcal {O}$-module, $H^*(\partial X_U,A)_\mathfrak {m}=0.$ They proved this using the fact that $\partial X_U$ admits a stratification with strata indexed by conjugacy classes of proper parabolic subgroups of $\textrm{GL}_n$ and by an in depth analysis of the cohomology of each stratum.

Recall that by [14, page 19], $M_\mu \otimes _\mathcal {O}\mathcal {O}/\varpi =M_{\mu ,\mathcal {O}/\varpi },$ which receives an action of $\textrm{GL}_n(\mathcal {O}/\varpi )$, compatible with that of $\textrm{GL}_n(\mathcal {O})$. Thus $M_{\mu ,\mathcal {O}/\varpi }$ is a smooth $\mathcal {O}[U_S]$-module that is finite as $\mathcal {O}$-module, so

$$\begin{aligned} H^*(\partial X_U,M_{\mu ,\mathcal {O}/\varpi })_\mathfrak {m}=0. \end{aligned}$$

We get the desired result by considering the long exact sequence associated to

$$\begin{aligned} 0\rightarrow M_\mu \xrightarrow {\varpi } M_\mu \rightarrow M_{\mu ,\mathcal {O}/\varpi } \rightarrow 0 \end{aligned}$$

and applying the Nakayama lemma to the finite $\mathcal {O}$-module $H^*(\partial X_U,M_{\mu })_\mathfrak {m}=0.$

Part (b) now follows from the long exact sequence

$$\begin{aligned} \dots \rightarrow H^i_c(X_U,M_\mu ) \rightarrow H^i(X_U,M_\mu ) \rightarrow H^i(\partial X_U,M_\mu )\rightarrow \dots \end{aligned}$$

For part (c), note that $T^S(H)_\mathfrak {m}/\mathcal {O}\text {-tors}\cong \mathbb {T}^S(\overline{H_\mathfrak {m}})$ for $H\in \{H^*(X_U,M_{\mu }),H^*_!(X_U,M_{\mu })\}$ by the same proof as Remark 4.3

For part (d), it is proved in [7, Theorem 2.4.10] using Franke’s decomposition of $H^*(X_U,M_{\mu ,\mathbb {C}})$ via automorphic forms that $H^*(X_U,M_{\mu ,\mathbb {C}})_\mathfrak {m}=H^*_{cusp}(X_U,M_{\mu ,\mathbb {C}})_\mathfrak {m}.$ As $H^*_{!}(X_U,M_{\mu ,\mathbb {C}})_\mathfrak {m}$ is always sandwiched between these two groups, these groups are all equal. (The first equality also follows from part (b).) $\square$

For readers’ convenience, we restate our running assumptions.

#### Corollary 4.20

Let the notation be as at the start of Section 4. Suppose in addition that *F* is a CM field that contains an imaginary quadratic field, and *S* comes via pullback from a set of finite places of $\mathbb {Q}$ which contains *p* and all places at which $F/\mathbb {Q}$ is ramified.

Assume $\mathfrak {m}$ is non-Eisenstein. Consider the following two statements:

- 28
  $$\begin{aligned} \varpi \mid L^{alg}(1,\pi ,{{\,\textrm{Ad}\,}}^0). \end{aligned}$$
- There is a cohomological cuspidal automorphic representation $\pi '\not \cong \pi$ of weight $\iota \mu$ of $\textrm{GL}_n(\mathbb {A}_F)$ with $(\pi ')^U\ne 0$ whose Hecke eigensystem $\Lambda ':\mathbb {T}^S\rightarrow \overline{\mathbb {Q}}_p$ satisfies $|\Lambda (t)-\Lambda '(t)|_p<1$ for all $t\in \mathbb {T}^S$.

Then (a) implies (b). If $H^b_!(X_U,M_{\mu ,\mathcal {O}})_\mathfrak {m}[\epsilon ]/\mathcal {O}\text {-torsion}$ is a free *T*-module, then (b) implies (a).

#### Proof

The proof is just a slight variation of that of Corollary 4.17. Abusing notation, we shall identify $\overline{\mathbb {Q}}_p$ with $\mathbb {C}$ using our fixed isomorphism $\iota$. Suppose $\varpi \mid L^{alg}(1,\pi ,{{\,\textrm{Ad}\,}}^0,\epsilon )$. By Theorem 4.14 and Lemma 3.4, $\eta _\pi \ne \mathcal {O}$. By Remark 3.7 there exists a $\overline{\mathbb {Q}}_p$-algebra homomorphism $\lambda ':T\otimes _\mathcal {O}\overline{\mathbb {Q}}_p \rightarrow \overline{\mathbb {Q}}_p$ such that $\lambda '\ne \lambda \otimes _{\mathcal {O}}\overline{\mathbb {Q}}_p$ and $|\lambda (t)-\lambda '(t)|<1$ for all $t\in T$. We have a natural map

$$\begin{aligned} \mathbb {T}^S_{\overline{\mathbb {Q}}_p}(V)\rightarrow T\otimes _\mathcal {O}\overline{\mathbb {Q}}_p=\mathbb {T}^S_{\bar{\mathbb {Q}}_p}(H^*_{cusp}(X_U,M_{\mu ,\bar{\mathbb {Q}}_p})_\mathfrak {m}), \end{aligned}$$

where the last equality is by Lemma 4.19, $V:=H^*_{cusp}(X_U,M_{\mu ,\overline{\mathbb {Q}}_p})$, and $\mathbb {T}^S_{\overline{\mathbb {Q}}_p}(V):= im(\mathbb {T}^S\otimes _\mathcal {O}\bar{\mathbb {Q}}_p \rightarrow {{\,\textrm{End}\,}}_{\bar{\mathbb {Q}}_p}(V))$. Composing this with $\lambda '$, we get a $\overline{\mathbb {Q}}_p$-algebra homomorphism

$$\begin{aligned} f:\mathbb {T}^S_{\overline{\mathbb {Q}}_p}(V)\rightarrow \overline{\mathbb {Q}}_p. \end{aligned}$$

Let $$\mathfrak {n}:=\ker f$$. Then

$$\begin{aligned} V_\mathfrak {n}\ne 0 \end{aligned}$$

because $Supp_{\mathbb {T}^S_{\overline{\mathbb {Q}}_p}(V)}(V)= \{\mathfrak p\in Spec(\mathbb {T}^S_{\overline{\mathbb {Q}}_p}(V)): \mathfrak p\supset Ann_{\mathbb {T}^S_{\overline{\mathbb {Q}}_p}(V)}(V)\}=Spec(\mathbb {T}^S_{\overline{\mathbb {Q}}_p}(V)).$ By [19, section 2.5.1], $V[\mathfrak {n}]\ne 0$. Thus, there is a cohomological cuspidal automorphic representation $\pi '$ of weight $\iota \mu$ such that $(\pi '^{\infty ,S})^{U^S}$ is isomorphic to a sub-$\mathbb {T}^S_{\overline{\mathbb {Q}}_p}(V)$-module of *V* and $(\pi '^{\infty ,S})^{U^S}[\mathfrak {n}]\ne 0.$ Note that $(\pi '^{\infty ,S})^{U^S}= \otimes '_v (\pi '_v)^{U_v}$ is one dimensional over $\overline{\mathbb {Q}}_p$, so $(\pi '^{\infty ,S})^{U^S}[\mathfrak {n}]= (\pi '^{\infty ,S})^{U^S}.$ The Hecke eigensystem attached to $\pi '$ has the desired property.

When $H^b_!(X_U,M_{\mu ,\mathcal {O}})_\mathfrak {m}[\epsilon ]/\mathcal {O}\text {-torsion}$ is a free *T*-module, the converse follows from Corollary 4.17 because if $\pi '$ is a cohomological cuspidal automorphic representation $\pi '$ of weight $\iota \mu$ of $\textrm{GL}_n(\mathbb {A}_F)$ with $(\pi ')^U\ne 0$, then $\pi '$ is a discrete automorphic representation and $0\ne H^b_{cusp}(X_U,M_{\mu ,\mathbb {C}})[(\pi '^{\infty ,S})^{U^S}] \subset H^b_!(X_U,M_{\mu ,\mathbb {C}})[(\pi '^{\infty ,S})^{U^S}]$. $\square$

### Selmer groups

We now illustrate how to combine the results above with deformation theory to obtain some Bloch-Kato type results relating Selmer groups and *L*-functions. We use the same definitions and notation of local and global deformation problems as in [7, section 6.2.1] and we will always take $\Lambda _v=\mathcal {O}$ for all $v\in S$. In particular, $\bar{\rho }:G_{F,S}\rightarrow \textrm{GL}_n(k)$ is absolutely irreducible, $\mathcal {D}_v$ is a local deformation problem for each $v\in S$,

$$\begin{aligned} \mathcal {S}=(\bar{\rho },S,\{\mathcal {O}\}_{v\in S},\{\mathcal {D}_v\}_{v\in S}) \end{aligned}$$

is a global deformation problem, and $R_{\mathcal {S}}$ is the ring representing the deformation functor of type $\mathcal {S}$.

Fix $\rho :G_{F,S} \rightarrow \textrm{GL}_n(\mathcal {O})$ a lifting of $\bar{\rho }$ of type $\mathcal {S}$. For each $m\ge 1$, let

$$\begin{aligned} \mathcal {O}_m:= \mathcal {O}\oplus \frac{\pi ^{-m}\mathcal {O}}{\mathcal {O}} \epsilon \end{aligned}$$

with multiplication given by $(a,b\epsilon )(c,d\epsilon )=(ac,(bc+ad)\epsilon )$. This is a local $\mathcal {O}$-algebra and there is a natural map $\mathcal {O}_m\twoheadrightarrow \mathcal {O}$ given by projection to the first factor.

#### Definition 4.21

We let

$$\begin{aligned} \mathcal {L}^1_v(\pi ^{-m}\mathcal {O}/\mathcal {O}) \end{aligned}$$

be the preimage of $\mathcal {D}_v(\mathcal {O}_m)$ under the isomorphism

$$Z^1\left( G_{F_v},{{\,\textrm{Ad}\,}}\rho \otimes _\mathcal {O}\frac{\pi ^{-m}\mathcal {O}}{\mathcal {O}}\right) \xrightarrow {\sim } \{\text {liftings } G_{F_v}\rightarrow \textrm{GL}_n(\mathcal {O}_m)\text { of }\rho |_{G_{F_v}}\}$$

given by $c\mapsto (1+c\epsilon )\rho |_{G_{F_v}},$ where $Z^1$ means the group of continuous 1-cocycles.

We know

15 $$\begin{aligned} Z^1(G_{F_v},{{\,\textrm{Ad}\,}}\rho \otimes _\mathcal {O}E/\mathcal {O})\xrightarrow {\sim } \{\text {liftings } G_{F_v}\rightarrow \textrm{GL}_n(\mathcal {O}\oplus \dfrac{E}{\mathcal {O}}\epsilon )\text { of }\rho |_{G_{F_v}}\} \end{aligned}$$

Since^29^$Z^1(G_{F_v},{{\,\textrm{Ad}\,}}\rho \otimes _\mathcal {O}E/\mathcal {O}) = \varinjlim _m Z^1(G_{F_v},{{\,\textrm{Ad}\,}}\rho \otimes _\mathcal {O}\frac{\pi ^{-m}\mathcal {O}}{\mathcal {O}})$, any lifting $G_{F_v}\rightarrow \textrm{GL}_n(\mathcal {O}\oplus \dfrac{E}{\mathcal {O}}\epsilon )\text { of }\rho |_{G_{F_v}}$ necessarily has image in $\textrm{GL}_n(\mathcal {O}_m)$ for some $m\ge 1$.

#### Definition 4.22

30 A lifting $G_{F_v}\rightarrow \textrm{GL}_n(\mathcal {O}\oplus \dfrac{E}{\mathcal {O}}\epsilon )\text { of }\rho |_{G_{F_v}}$ is of type $\mathcal {D}_v$ if it is of type $\mathcal {D}_v$ when it is regarded as a lift with codomain in $\textrm{GL}_n(\mathcal {O}_m)$ for some *m* (or, equivalently, for all *m* for which $\textrm{GL}_n(\mathcal {O}_m)$ contains the image of the lift.)

The following is immediate from the definitions.

#### Definition/Lemma 4.23

The following subgroups of $Z^1(G_{F_v},{{\,\textrm{Ad}\,}}\rho \otimes _\mathcal {O}E/\mathcal {O})$ are equal. We denote them by $\mathcal {L}^1_v(E/\mathcal {O})$.

(i) $$\varinjlim _m \mathcal {L}^1_v(\pi ^{-m}\mathcal {O}/\mathcal {O})$$
(ii) preimage of $\{\text {liftings } G_{F_v}\rightarrow \textrm{GL}_n(\mathcal {O}\oplus \dfrac{E}{\mathcal {O}}\epsilon )\text { of }\rho |_{G_{F_v}}\text { of type }\mathcal {D}_v\}$ under the isomorphism (15).

Since $\rho |_{G_{F_v}}$ is of type $\mathcal {D}_v$, $a\rho |_{G_{F_v}} a^{-1}$ is also of type $\mathcal {D}_v$ for all $a\in \ker (\textrm{GL}_n(\mathcal {O}_m)\rightarrow \textrm{GL}_n(\mathcal {O}))$ for all *m* by definition of local deformation problem. It follows that $\mathcal {L}^1_v(\pi ^{-m}\mathcal {O}/\mathcal {O})$ and $\mathcal {L}^1_v(E/\mathcal {O})$ both contain the group of 1-boundaries.

#### Definition 4.24

We define $\mathcal {L}_v(\pi ^{-m}\mathcal {O}/\mathcal {O})$ to be the image of $\mathcal {L}^1_v(\pi ^{-m}\mathcal {O}/\mathcal {O})$ under $Z^1\rightarrow H^1$. Similarly, we define $\mathcal {L}_v(E/\mathcal {O})$ to be the image of $\mathcal {L}^1_v(E/\mathcal {O})$ under $Z^1\rightarrow H^1$. Equivalently, by exactness of direct limits, $\mathcal {L}_v(E/\mathcal {O})=\varinjlim _m \mathcal {L}_v(\pi ^{-m}\mathcal {O}/\mathcal {O}).$

We also define the Selmer groups

$$\begin{aligned} H^1_{\mathcal {S}}\left( {{\,\textrm{Ad}\,}}\rho \otimes _\mathcal {O}\frac{\pi ^{-m}\mathcal {O}}{\mathcal {O}}\right) := \left\{ c\in H^1\left( G_{F,S},{{\,\textrm{Ad}\,}}\rho \otimes _\mathcal {O}\frac{\pi ^{-m}\mathcal {O}}{\mathcal {O}}\right) :c_v\in \mathcal {L}_v(\pi ^{-m}\mathcal {O}/\mathcal {O})\;\forall v\in S\right\} \end{aligned}$$

and

$$\begin{aligned} H^1_{\mathcal {S}}\left( {{\,\textrm{Ad}\,}}\rho \otimes _\mathcal {O}\frac{E}{\mathcal {O}}\right) := \left\{ c\in H^1\left( G_{F,S},{{\,\textrm{Ad}\,}}\rho \otimes _\mathcal {O}\frac{E}{\mathcal {O}}\right) :c_v\in \mathcal {L}_v(E/\mathcal {O})\;\forall v\in S\right\} , \end{aligned}$$

where $c_v$ is the restriction of *c* to $G_{F_v}$. It is easy to verify that

$$\begin{aligned} H^1_{\mathcal {S}}\left( {{\,\textrm{Ad}\,}}\rho \otimes _\mathcal {O}\frac{E}{\mathcal {O}}\right) = \varinjlim _m H^1_{\mathcal {S}}\left( {{\,\textrm{Ad}\,}}\rho \otimes _\mathcal {O}\frac{\pi ^{-m}\mathcal {O}}{\mathcal {O}}\right) . \end{aligned}$$

#### Lemma 4.25

The strict equivalence class $[\rho ]$ of $\rho$ gives rise to a local $\mathcal {O}$-algebra homomorphism $R_{\mathcal {S}}\xrightarrow {\theta } \mathcal {O}$. Let $\mathfrak p:=\ker \theta$. Then

$$\begin{aligned} {{\,\textrm{Hom}\,}}_\mathcal {O}(\mathfrak p/\mathfrak p^2, E/O) \cong H^1_{\mathcal {S}}({{\,\textrm{Ad}\,}}\rho \otimes _\mathcal {O}E/\mathcal {O}), \end{aligned}$$

#### Proof

This is well-known so we will just sketch a proof. Note that $\mathfrak p/\mathfrak p^2$ is a finitely generated $R_{\mathcal {S}}/\mathfrak p=\mathcal {O}$-module, so any $\mathcal {O}$-algebra homomorphism $\mathfrak p/\mathfrak p^2 \rightarrow E/\mathcal {O}$ has image contained in $\varpi ^{-m}\mathcal {O}/\mathcal {O}$ for some $m\ge 1$. Thus, if we know

$$\begin{aligned} {{\,\textrm{Hom}\,}}_\mathcal {O}(\mathfrak p/\mathfrak p^2, \varpi ^{-m}\mathcal {O}/\mathcal {O}) \cong H^1_{\mathcal {S}}({{\,\textrm{Ad}\,}}\rho \otimes _\mathcal {O}\varpi ^{-m}\mathcal {O}/\mathcal {O}) \end{aligned}$$

for all $m\ge 1$, then taking colimit will give the desired result. This follows from the chain of isomorphisms

16 $$\begin{aligned}&\;\;\;H^1_{\mathcal {S}}({{\,\textrm{Ad}\,}}\rho \otimes _\mathcal {O}\varpi ^{-m}\mathcal {O}/\mathcal {O})\nonumber \\ \cong&\;\{\text {liftings } G_{F,S} \rightarrow \textrm{GL}_n(\mathcal {O}_m) \text { of }\rho \text { of type }\mathcal {S}\}/\ker (\textrm{GL}_n(\mathcal {O}_m)\rightarrow \textrm{GL}_n(\mathcal {O})) \end{aligned}$$

17 $$\begin{aligned} \cong&\;\{\text {deformations } G_{F,S} \rightarrow \textrm{GL}_n(\mathcal {O}_m) \text { of }[\rho ]\text { of type }\mathcal {S}\}\nonumber \\ \cong&\;\{f\in {{\,\textrm{Hom}\,}}_\mathcal {O}(R_{\mathcal {S}},\mathcal {O}_m): f\pmod \epsilon =\theta \}\nonumber \\ \cong&\;{{\,\textrm{Hom}\,}}_\mathcal {O}(\mathfrak p/\mathfrak p^2, \varpi ^{-m}\mathcal {O}/\mathcal {O}). \end{aligned}$$

In (16), $\ker (\textrm{GL}_n(\mathcal {O}_m)\rightarrow \textrm{GL}_n(\mathcal {O}))$ acts on $\{\text {liftings } G_{F,S} \rightarrow \textrm{GL}_n(\mathcal {O}_m) \text { of }\rho \text { of type }\mathcal {S}\}$ by conjugation. In (17), by deformations of $[\rho ]$, we mean deformations of $\bar{\rho }$ whose pushforward to $\textrm{GL}_n(\mathcal {O})$ is $[\rho ]$. To show the bijectivity of (16) and (17), note that the centralizer of the image of $\rho$ in $\textrm{GL}_n(\mathcal {O})$ is $\mathcal {O}^\times$ by [28, lemma 2.1.8] and $\bar{\rho }$ is absolutely irreducible. The other steps are easy. $\square$

#### Theorem 4.26

Let the notation be as at the start of Section 4. Suppose in addition that *F* is a CM field that contains an imaginary quadratic field, and *S* comes via pullback from a set of finite places of $\mathbb {Q}$ which contains *p* and all places at which $F/\mathbb {Q}$ is ramified.

Assume $\mathfrak {m}$ is non-Eisenstein. Then there is a continuous Galois representation

$$\begin{aligned} \rho _\mathfrak {m}:G_{F,S} \rightarrow \textrm{GL}_n(T) \end{aligned}$$

such that for all $v\notin S$ of *F*, the characteristic polynomial of $\rho _m({{\,\textrm{Frob}\,}}_v)$ is

$$\begin{aligned} X^n-T_{v,1}X^{n-1}+\dots +(-1)^i q_v^{i(i-1)/2}T_{v,i}X^{n-i}+\dots + (-1)^n q_v^{n(n-1)/2}T_{v,n}, \end{aligned}$$

where $T_{v,i}=[\textrm{GL}_n(\mathcal {O}_{F_v})diag(\varpi _v,\dots ,\varpi _v,1,\dots ,1)\textrm{GL}_n(\mathcal {O}_{F_v})]$ with $\varpi _v$ appearing *i* times and $T:=\mathbb {T}^S(H^*(X_U,M_{\mu }))_\mathfrak {m}/\mathcal {O}\text {-tors}$ as in Lemma 4.19.

Assume that $\rho _\mathfrak {m}$ is a lifting of $\bar{\rho }_\mathfrak {m}$ of type $\mathcal {S}$, where $\mathcal {S}=(\bar{\rho }_\mathfrak {m},S,\{\mathcal {O}\}_{v\in S},\{\mathcal {D}_v\}_{v\in S})$ is some global deformation problem. Let $\rho :=\lambda \circ \rho _\mathfrak {m}$, where $\lambda :T\rightarrow \mathcal {O}$ is induced from $\Lambda$ as in Lemma 4.2. Then^31^

18 $$\begin{aligned} \#H^1_{\mathcal {S}}({{\,\textrm{Ad}\,}}\rho \otimes _\mathcal {O}E/\mathcal {O}) \ge \# (\mathcal {O}/L^{alg}(1,\pi ,{{\,\textrm{Ad}\,}}^\circ )) \end{aligned}$$

where $\#$ denotes the order of a group.

#### Proof

By [7, theorem 2.4.10 (1)] or Lemma 4.1, $\lambda$ factors through the quotient $\mathbb {T}^S \rightarrow \mathbb {T}^S(R\Gamma (X_U,M_\mu ))$, so $\mathfrak {m}$ is the preimage of a maximal ideal $\mathfrak {n}$ of $\mathbb {T}^S(R\Gamma (X_U,M_\mu ))$. Clearly $\mathbb {T}^S(R\Gamma (X_U,M_\mu ))_\mathfrak {n}=\mathbb {T}^S(R\Gamma (X_U,M_\mu ))_\mathfrak {m}.$ By [7, theorem 2.3.7], there is a nilpotent ideal $I\subset \mathbb {T}^S(R\Gamma (X_U,M_\mu ))$ and a continuous Galois representation

$$\begin{aligned} G_{F,S} \rightarrow \textrm{GL}_n(\mathbb {T}^S(R\Gamma (X_U,M_\mu ))_\mathfrak {n}/I) \end{aligned}$$

such that for all $v\notin S$ of *F*, the characteristic polynomial of the image of ${{\,\textrm{Frob}\,}}_v$ is

$$\begin{aligned} X^n-T_{v,1}X^{n-1}+\dots +(-1)^i q_v^{i(i-1)/2}T_{v,i}X^{n-i}+\dots + (-1)^n q_v^{n(n-1)/2}T_{v,n}. \end{aligned}$$

Composing this with the natural map^32^$\mathbb {T}^S(R\Gamma (X_U,M_\mu ))_\mathfrak {n}/I =\mathbb {T}^S(R\Gamma (X_U,M_\mu ))_\mathfrak {m}/I \twoheadrightarrow T$ gives $\rho _m$.

Note that *T* is a complete Noetherian local $\mathcal {O}$-algebra with residue field $k:=\mathcal {O}/\varpi$. By assumption, $\rho _\mathfrak {m}$ is of type $\mathcal {S}$, so its strict equivalence class induces an $\mathcal {O}$-algebra homomorphism $f:R_{\mathcal {S}}\rightarrow T$. We know that $\mathbb {T}^S$ is generated by

$$\begin{aligned} \{T^i_v,(T^n_v)^{-1}: v\notin S, 1\le i\le n\} \end{aligned}$$

as an $\mathcal {O}$-algebra^33^. For all $g\in G_{F,S}$, every coefficient of the characteristic polynomial of $\rho _m(g)$ is in the image of $R_{\mathcal {S}}\rightarrow T$. Taking ${{\,\textrm{Frob}\,}}_v^{\pm 1}$, we know *f* is surjective.

If $H^1_{\mathcal {S}}({{\,\textrm{Ad}\,}}\rho \otimes _\mathcal {O}E/\mathcal {O})$ is infinite, then equation (18) is trivial. Suppose $H^1_{\mathcal {S}}({{\,\textrm{Ad}\,}}\rho \otimes _\mathcal {O}E/\mathcal {O})$ is finite. Let $\theta =\lambda \circ f$ and $\mathfrak p:=\ker \theta$. It follows from Lemma 4.25 that $\mathfrak p/\mathfrak p^2$ is also finite and

$$\begin{aligned} \#H^1_{\mathcal {S}}({{\,\textrm{Ad}\,}}\rho \otimes _\mathcal {O}E/\mathcal {O}) = \#(\mathfrak p/\mathfrak p^2). \end{aligned}$$

By [29, page 141 equation (5.2.3)],

$$\begin{aligned} \#(\mathfrak p/\mathfrak p^2) \ge \#(\mathcal {O}/\eta _{R_{\mathcal {S}}}) \end{aligned}$$

where $\eta _{R_{\mathcal {S}}}:=\theta (Ann_{R_{\mathcal {S}}}(\mathfrak p))$. By [29, page 140 equation (5.2.2)],

$$\begin{aligned} \#(\mathcal {O}/\eta _{R_{\mathcal {S}}}) \ge \#(\mathcal {O}/\eta _T), \end{aligned}$$

where $\eta _T=\lambda (Ann_T(\ker \lambda ))$, which is the same as $\eta _\pi$ in the previous subsections. By Lemma 3.4, Lemma 4.19, and Theorem 4.14,

$$\begin{aligned} \#(\mathcal {O}/\eta _\pi ) \ge \#(\mathcal {O}/\eta _{\pi ,b,\epsilon }) \ge \# (\mathcal {O}/L^{alg}(1,\pi ,{{\,\textrm{Ad}\,}}^\circ ,\epsilon )). \end{aligned}$$

We get the desired inequality. $\square$

As an illustration of Theorem 4.26, let us give an example. Let $\mathcal {D}_v^\Box$ be the functor on $CNL_\mathcal {O}$ (category of complete Noetherian local $\mathcal {O}$-algebras with residue fields *k*) that sends *A* to the set of all lifts of $\bar{\rho }_\mathfrak {m}|_{G_{F_v}}$ to *A*. In the Fontaine-Laffaille case, if *v* is a *p*-adic place, we let $\mathcal {D}_v^{FL}$ be the local deformation problem that sends any $A\in CNL_\mathcal {O}$ that is finite over $\mathcal {O}$ to all liftings of $\bar{\rho }_\mathfrak {m}|_{G_{F_v}}$ to *A* that are Fontaine-Laffaille of type $(\mu _\tau )_{\tau \in Hom(F_v,E)}$. See [7, sections 4.1, 6.2.14] for more detail.

#### Corollary 4.27

Let the notation be as at the start of Section 4 with *F* CM. Suppose in addition that

- *F* contains an imaginary quadratic field in which *p* splits
- *S* comes via pullback from a set of finite places of $\mathbb {Q}$ which contains *p* and all places at which $F/\mathbb {Q}$ is ramified
- $$U_v=\textrm{GL}_n(\mathcal {O}_{F_v})$$ for all $$v\mid p$$.
- *p* is unramified in *F*
- For each embedding $\tau :F\hookrightarrow \bar{\mathbb {Q}}_p$, we have
  $$\begin{aligned} \mu _{\tau ,1}+\mu _{\tau c,1}-\mu _{\tau ,n}-\mu _{\tau c,n} < p-2n. \end{aligned}$$
- For each $v\mid p$, let $\bar{v}=v|_{F^+}$. Then there is a *p*-adic place $\bar{v}'\ne \bar{v}$ of $F^+$ such that
  $$\begin{aligned} \sum _{\bar{v}''\ne \bar{v},\bar{v}'} [F_{\bar{v}''}:\mathbb {Q}_p] > \frac{1}{2}[F^+:\mathbb {Q}]. \end{aligned}$$
- $\mathfrak {m}$ is non-Eisenstein
- $$p>n^2$$
- the residual Galois representation is decomposed generic [7, Definition 4.3.1].

Then Theorem 4.26 holds with $\mathcal {S}=(\bar{\rho }_\mathfrak {m},S,\{\mathcal {O}\}_{v\in S}, \{\mathcal {D}_v^{FL}\}_{v\mid p}\cup \{\mathcal {D}_v^\Box \}_{v\in S-\{v\mid p\}}).$

#### Proof

This follows immediately from [7, Theorem 4.5.1]. $\square$

#### Remark 4.28

To relate this Selmer group to the Bloch-Kato Selmer group, see [30, lemma 2.1] and [31, section 7.3]. In their setup, the $\mathcal {O}$-Fitting ideal of their Selmer group was equal to that of the Bloch-Kato one multiplied by $\prod _{v\in \Sigma }Fitt_\mathcal {O}(H^1_f(F_v,({{\,\textrm{Ad}\,}}^\circ \rho _f)^*(1)))$ for some finite set of places $\Sigma$. Each term in the product was then shown to be equal to the local Tamagawa number divided by the local *L*-factor at that place. Combining this with a suitable $R=T$ theorem, they were able to deduce a form of the Bloch-Kato conjecture using similar argument to the proof of Theorem 4.26.

## Data Availability

We use no data in this paper.

## Footnotes

1 $A_G=\mathbb {R}_{>0}$ is embedded diagonally into the centre of $G(F_\infty ).$

2 We quotient out by $K_\infty$ rather than $K_\infty ^\circ$ because we would like to realise cohomological automorphic representations in cohomology. If we instead quotient out by $K_\infty$, then some of these representations only appear in the relative Lie algebra after twisting by a character $\epsilon$ of $\widehat{K_\infty /K_\infty ^\circ }$, but the fact that $\epsilon$ is not a character of $U\subset G(\mathbb {A}_F^\infty )$ makes it unclear how to define the inner cohomology with coefficient in $M_\lambda \otimes \epsilon$. See however [12, Proposition 3.2.5, Theorem 4.3.5] for a possible way to do this using locally algebraic representations.

3 In [10], this is stated for good subgroup, but the proof actually works for all neat subgroups.

4 If we write the characters of $T_n$ as $\lambda =(\lambda _1,\dots ,\lambda _n)$, then $w_0\lambda =(\lambda _n,\dots ,\lambda _1)$

5 Here, $\mathbb {A}^1_A={{\,\textrm{Spec}\,}}A[T]$ is the affine line over *A*.

6 Strictly speaking, the formalism gives us an action of $\mathcal {H}(\textrm{GL}_n(\mathbb {A}_F^{\infty ,p})\times U_p,U).$ Yet, this algebra is canonically isomorphic to $\mathcal {H}(\textrm{GL}_n(\mathbb {A}_F^{\infty ,p}),U^p)$

7 Recall that $A_G=\mathbb {R}_{>0}$ is embedded diagonally into the centre of $G(F_\infty ).$

8 For instance, we can always twist $\pi$ by a Hecke character such that the action of $A_G$ is trivial, i.e. $\chi =1$. This reduces us to the case in [15, section 3.7].

9 This is *C*-algebraic in the sense of Buzzard-Gee.

10 It is also common in the literature to write $q_0$ for $b_n$ and $q_0+\ell _0$ for $t_n$.

11 This means the two induced maps $M_1\rightarrow {{\,\textrm{Hom}\,}}_\mathcal {O}(M_2,\mathcal {O})$ and $M_1\rightarrow {{\,\textrm{Hom}\,}}_\mathcal {O}(M_2,\mathcal {O})$ are isomorphisms.

12 We also write $[,\,]$ for its extension $(M_1)_E\times (M_2)_E \rightarrow E$.

13 Such a *U* always exists by the uniqueness of local new vector.

14 $\mathbb {Q}(\pi )$ is the field of rationality of $\pi$ [9, section 3 page 101].

15 Note that since $\mathcal {O}$ is a PID, an $\mathcal {O}$-module is finite flat iff it is finite free.

16 Such a $\psi$ exists, e.g. take $\psi _v(x)=e^{-2\pi ix}$ for real *v*, $\psi _v(x)=e^{-2\pi i(x+\bar{x})}$ for complex *v*, and $\psi _v(x)=e^{2\pi i Tr_{F_v/\mathbb {Q}_p}(x)}$ for all $v\mid p$ and all rational primes *p*, and $\psi =\prod _v \psi _v$.

17 Well-defined by the multiplicity one theorem.

18 If $\pi _v$ is unramified, then $w_v$ is the unique element in $W(\pi _v,\psi _v)^{\textrm{GL}_n(\mathcal {O}_{F_v})}$ with $w_v(I_n)=1$.

19 This is a homomorphism because $\mathcal {H}^S(G^S,U^S)$ is commutative.

20 In general, $M_\mu ^\vee$ and $M_{\mu ^\vee }$ are *not* isomorphic.

21 By ’induces’, we mean the map given by lifting an element of $\mathbb {T}^S(\overline{H}^b_!(X_U,M_\mu )_\mathfrak {m})$ to $\mathbb {T}^S$, applying $\theta$ to it, and then projecting it to $\mathbb {T}^S(\overline{H}^t_!(X_U,M_\mu ^\vee )_{\tilde{\mathfrak {m}}}).$

22 However, our $L^{alg}(1,\pi ,{{\,\textrm{Ad}\,}}^0,\epsilon )$ and periods are slightly different from that in [1] due to our different choice of $X_U$.

23 This is the only place in this proof where we use the assumption that $H^b(\partial X_U,M_\mu )_\mathfrak {m}$ is $\mathcal {O}$-torsion free.

24 The reason that we need to argue via $e', \tilde{e}'$ rather than $e,\tilde{e}$ directly is that we do not have the analogue of part (b) of Corollary 4.10 for the entire inner cohomology. We only have it for the bottom and top degrees.

25 At least in the totally real case, although it was not explicitly stated, they implicitly assumed that the ideal $\mathfrak {m}$ is non-Eisenstein, as they applied the Poincaré duality result from [4, Proposition 4.10 part 3], which was established only under this assumption in their paper.

26 This is defined using the fixed isomorphism $\iota :\overline{\mathbb {Q}}_p\xrightarrow {\sim } \mathbb {C}$ as in Lemma 4.1. The only differences are that $(\pi '^{\infty ,S})^{U^S}$ only appears in the inner cohomology but not the cuspidal cohomology, and the image of $\Lambda '$ needs not lie in $\mathcal {O}$, but only an integral extension.

27 We think that in that (well-written) paper, it is necessary for $p\ge 7$ instead of $p\ge 3$ as stated. This ensures the image of the residual Galois representation is enormous, guaranteeing the existence of Taylor-Wiles primes. Also, it seems that for the first equation on page 8 to hold, one should patch the (derived) dual of $C^\bullet _n$ rather than $C^\bullet _n$ itself.

28 Since *F* is CM, $K_\infty /K_\infty ^\circ$ is trivial. There is therefore no need to choose $\epsilon \in \widehat{K_\infty /K_\infty ^\circ }$, so we omit it from $L^{alg}(1,\pi ,{{\,\textrm{Ad}\,}}^0,\epsilon )$.

29 Note that ${{\,\textrm{Ad}\,}}\rho \otimes _\mathcal {O}E/\mathcal {O}$ is a discrete $G_{F_v}$-module.

30 Since $\mathcal {O}\oplus \frac{E}{\mathcal {O}} \epsilon$ is not a complete local ring, the term ’of type $\mathcal {D}_v$’ is not defined for liftings to $\textrm{GL}_n(\mathcal {O}\oplus \frac{E}{\mathcal {O}} \epsilon )$ a priori.

31 Recall that since *F* is CM, there is no need to choose $\epsilon \in \widehat{K_\infty /K_\infty ^\circ }$ and we write $L^{alg}(1,\pi ,{{\,\textrm{Ad}\,}}^\circ )$ for $L^{alg}(1,\pi ,{{\,\textrm{Ad}\,}}^\circ ,1)$.

32 This map exists because *T* is reduced by Lemma 4.2.

33 This can be easily deduced by applying the Satake isomorphism to $\mathcal {H}(\textrm{GL}_n(F_v),\textrm{GL}_n(\mathcal {O}_{F_v}))\otimes _\mathbb {Z}\mathbb {Z}[q_v^{1/2}]$.
